# Poly(DADMAC) incorporated lipid nanoparticles enhance the delivery of antimicrobial peptides into plant cells

**DOI:** 10.1038/s41598-026-52008-6

**Published:** 2026-05-13

**Authors:** Samavath Mallawarachchi, Gayan Ivantha Nawaratna, Stanislav Vitha, Nalin Samarasinghe, Gaya P. Yadav, Kranthi Mandadi, James Borneman, Sandun Fernando

**Affiliations:** 1 Department of Biological and Agricultural Engineering, Texas A&M University, College Station, TX 77843 USA; 2 Department of Biochemistry and Biophysics, Texas A&M University, College Station, TX 77843 USA; 3 Microscopy and Imaging Center, Texas A&M University, College Station, TX 77843 USA; 4 Texas A&M AgriLife Research & Extension Center, Weslaco, TX 78596 USA; 5 Department of Plant Pathology and Microbiology, Texas A&M University, College Station, TX 77843 USA; 6 Institute for Advancing Health Through Agriculture, Texas A&M AgriLife, TX College Station, USA; 7 Department of Microbiology & Plant Pathology, University of California-Riverside, Riverside, CA 92507 USA

**Keywords:** Poly(DADMAC), Cationic nanoparticles, Peptide delivery, Vascular delivery, Citrus greening, Biochemistry, Biological techniques, Biotechnology, Drug discovery, Nanoscience and technology

## Abstract

*Candidatus* Liberibacter asiaticus (CLas) is a phloem-limited bacterium that causes the citrus greening disease. One of the main challenges in treating citrus greening disease is delivering the therapeutic agents to the vascular system where CLas bacteria reside. This study proposes Polydiallyldimethylammonium chloride (poly(DADMAC)) incorporated lipid nanoparticles as a potential method to deliver the drugs into the plant vascular system via foliar application. The positive charge of poly(DADMAC) is expected to permeabilize the negatively charged cell surface, allowing therapeutic particles to permeate through the cell surface. Glyceryl monostearate-based lipid nanoparticles were synthesized using a high-shear homogenization method using Tween 80 and poly(DADMAC) as surfactants, and were characterized using a particle size analyzer. Nanoparticles containing 0.5% Tween 80 and 0.5% poly(DADMAC) displayed the most desirable characteristics, with an average particle size of 513.6 nm, an average zeta potential of + 13.78 mV, and an encapsulation efficiency of 94.8%. Successful encapsulation of anti-microbial peptides in the nanoparticles was verified using fluorescence imaging and fluorescence lifetime analysis. Fluorescence imaging of leaf vascular tissues showed significantly higher fluorescence in samples treated with nanoparticles encapsulating fluorescent-tagged peptides, compared to untreated samples and those treated with aqueous peptides. The presence of the peptide in nanoparticle-treated leaves was also validated using Matrix-Assisted Laser Desorption/Ionization – Time of Flight (MALDI-TOF) mass spectrometry. Thus, these results indicate that poly(DADMAC) incorporated nanoparticles can enhance the migration of peptides into vascular tissues. This can be an effective and convenient technique to deliver therapeutics against citrus greening disease.

**Supplementary Information:**

The online version contains supplementary material available at 10.1038/s41598-026-52008-6.

## Introduction

*Candidatus Liberibacter asiaticus* (CLas) is a fastidious, gram-negative bacterium and the main putative causative agent of Citrus greening disease, also known as Huanglongbing (HLB), which has caused extensive damage to citrus plantations worldwide^1,2^. This bacterium is phloem-limited and transmitted among plants via the psyllid vector *Diaphorina citri*^3–5^. The phloem-limited nature of this bacterium presents multiple challenges in mitigating the disease, including the difficulty of detection, the inability to culture the bacterium for drug screening purposes, and the difficulty of delivering therapeutic agents to the target cells^6–9^. Delivery of drugs into the vascular system is a challenge associated with many phloem-limited diseases, and it is reported that in most cases, only about 1% of the drugs applied to the leaves can penetrate the leaves and enter the vascular system^10,11^. Furthermore, inadequate absorption following foliar applications of therapies can lead to adverse environmental issues such as contamination of soil and water, and disruption of the soil microbiome^11,12^. Therefore, delivering therapeutic agents to the vascular tissues where the bacteria reside is a key focus in citrus greening-related research.

While several techniques such as precision injection, lipid nanoparticles, polymeric nanoparticles, and cell penetrating peptides, have been reported to effectively deliver some therapeutic molecules to plant cells, most of those techniques have not been used on a commercial scale^10,12–20^. Among these techniques, lipid nanoparticles have been widely used for many intracellular drug delivery applications, mostly in mammalian cells, since they have lower toxicity and higher biocompatibility compared to other cell penetration techniques, such as polymeric nanoparticles^21–27^. Other advantages of lipid nanoparticles include high site-specificity, formulation simplicity, and the feasibility of scale-up^23–25,28^. Multiple types of lipid nanoparticles have been engineered and used for drug delivery applications in recent years, including solid lipid nanoparticles (SLNs), nanostructured lipid carriers (NLCs), lipid-drug conjugates, and liposomes, with SLNs being the most commonly used^23,29–31^. Among lipid nanoparticles, cationic nanoparticles have been used most, since they can interact with the negatively charged cell membrane, and can achieve high loading of negatively charged drugs like RNA^24,32,33^. Cationic agents, which have been used to increase the positive charge of SLNs, include lipids such as *N*-[1-(2,3-Dioleoyloxy) propyl]-*N*,*N*,*N*-trimethylammoniumchloride (DOTAP), and surfactants such as stearylamine and cetyltrimethylammonium bromide^33–36^.

Quaternary ammonium compounds (QACs) are a group of compounds that have shown the ability to penetrate the cell wall. These compounds consist of a cationic amine group and a long hydrophobic group. Overall, the cationic nature of these compounds enables them to rapidly attach to the negatively charged cell surface, causing disruption or penetration of the cell wall and cell membrane^37–40^. Due to their ability to disrupt the cell surface, QACs have been widely used as antimicrobial agents^39,41–44^. QACs such as esterquat1 have also been successfully used in lipid nanoparticle formulations used for drug delivery^34,39,45^. However, when using QACs for drug delivery, the concentration of QAC needs to be carefully controlled, since high concentrations of QACs can lead to complete disruption of the cell wall, leading to toxicity and cell death^36,37^.

This study evaluates the potential of using Polydiallyldimethylammonium chloride (Poly(DADMAC)) for delivering anti-microbial peptides into the vascular tissues of citrus trees. Poly(DADMAC) is a QAC that has been successfully used to pierce algal cell walls^38^. Compared to other cationic polymers which have mainly been used for drug delivery into mammalian cells, poly(DADMAC) has been reported to permeabilize the thick cell walls of algal cells, which closely mimics the barriers encountered in plant vascular tissues. Due to its ability to permeabilize cell walls, incorporation of poly(DADMAC) is expected to facilitate the successful delivery of the peptides into the vascular system.

## Methods

### Materials

Peptide 57,020 (Sequence: SRNSSIALGLRRAYAVFNYFVARGI) with 5-carboxyfluorescein (FAM) fluorescent tag at N-terminus, was custom synthesized by Genscript, USA. This peptide possesses antimicrobial activity by binding to the bacterial outer membrane protein (BamA)^46^. Glyceryl monostearate, Tween 80, and Nile Red were purchased from VWR USA, while low molecular weight poly(DADMAC) (molecular weight < 100,000 g/mol) and propyl gallate were purchased from Sigma Aldrich. Meyer lemon (*Citrus meyeri*) plants used for the experiments were purchased from Plants N Things Nursery in Brenham, TX.

### Synthesis of nanoparticles

Poly(DADMAC) incorporated Solid Lipid Nanoparticles (SLNs) were synthesized using high shear homogenization as described previously^47–49^. Aqueous phase was prepared by adding measured amounts of Tween 80 and poly(DADMAC) to deionized water, keeping the total weight to 86 g. This aqueous phase was heated to 65 °C under constant stirring, while 10 g of Glyceryl monostearate lipid was separately heated to 65 °C. After the desired temperatures were reached, 4 mL of 1000 µg/mL peptide solution was added to the aqueous phase, followed by the melted lipid. This mixture was homogenized for 4 min at 6000 rpm, followed by 1 min at 10,000 rpm using Silverson L5M-A high shear homogenizer. The resulting suspension was refrigerated at 4 °C for 10 min to obtain the SLNs.

### Analysis of particle size and zeta potential

The size and zeta potential of SLNs were measured using a Beckman Coulter Delsa Nano C particle analyzer. Both parameters were measured using the flow cell, and the pinhole size was maintained at 50 nm. Size measurements were done using 70 accumulation times, and zeta measurements were done using 30 accumulation times at five different positions.

### Measuring encapsulation efficiency

Encapsulation efficiency is defined as the ratio between the amount of drug incorporated into SLNs and the total amount of drug introduced to the solution^47^. Initial peptide concentration of aqueous phase was determined by measuring the fluorescence intensity at emission wavelength of 520 nm and excitation wavelength of 490 nm, which corresponds to Fluorescein. Fluorescence intensities were measured using a Barnstead Thermolyne Quantech Fluorometer. Once the nanoparticle suspension was formed, 10 ml of the suspension was taken into a 15 ml centrifuge tube and centrifuged at 6000 rpm for 2 min. After 2 min, the supernatant was separated and subjected to two more centrifugation cycles. Fluorescence intensity of the final supernatant after 3 cycles at 500 nm was measured, and peptide concentration was determined using a calibration curve. Encapsulation efficiency and peptide loading were calculated using Eqs. 1 and 2.

1 $$\begin{aligned}\:\mathrm{E}\mathrm{n}\mathrm{c}\mathrm{a}\mathrm{p}\mathrm{s}\mathrm{u}\mathrm{l}\mathrm{a}\mathrm{t}\mathrm{i}\mathrm{o}\mathrm{n}\:\text{ }\mathrm{e}\mathrm{f}\mathrm{f}\mathrm{i}\mathrm{c}\mathrm{i}\mathrm{e}\mathrm{n}\mathrm{c}\mathrm{y}\:\\=\frac{\mathrm{P}\mathrm{e}\mathrm{p}\mathrm{t}\mathrm{i}\mathrm{d}\mathrm{e}\:\text{ }\mathrm{c}\mathrm{o}\mathrm{n}\mathrm{c}\mathrm{e}\mathrm{n}\mathrm{t}\mathrm{r}\mathrm{a}\mathrm{t}\mathrm{i}\mathrm{o}\mathrm{n}\:\text{ }\mathrm{o}\mathrm{f}\:\text{ }\mathrm{a}\mathrm{q}\mathrm{u}\mathrm{e}\mathrm{o}\mathrm{u}\mathrm{s}\:\text{ }\mathrm{p}\mathrm{h}\mathrm{a}\mathrm{s}\mathrm{e}\:\text{ }\mathrm{b}\mathrm{e}\mathrm{f}\mathrm{o}\mathrm{r}\mathrm{e}\:\text{ }\mathrm{m}\mathrm{i}\mathrm{x}\mathrm{i}\mathrm{n}\mathrm{g}-\mathrm{P}\mathrm{e}\mathrm{p}\mathrm{t}\mathrm{i}\mathrm{d}\mathrm{e}\:\text{ }\mathrm{c}\mathrm{o}\mathrm{n}\mathrm{c}\mathrm{e}\mathrm{n}\mathrm{t}\mathrm{r}\mathrm{a}\mathrm{t}\mathrm{i}\mathrm{o}\mathrm{n}\:\text{ }\mathrm{o}\mathrm{f}\:\text{ }\mathrm{a}\mathrm{q}\mathrm{u}\mathrm{e}\mathrm{o}\mathrm{u}\mathrm{s}\:\text{ }\mathrm{p}\mathrm{h}\mathrm{a}\mathrm{s}\mathrm{e}\:\text{ }\mathrm{a}\mathrm{f}\mathrm{t}\mathrm{e}\mathrm{r}\:\text{ }\mathrm{c}\mathrm{e}\mathrm{n}\mathrm{t}\mathrm{r}\mathrm{i}\mathrm{f}\mathrm{u}\mathrm{g}\mathrm{a}\mathrm{t}\mathrm{i}\mathrm{o}\mathrm{n}}{\mathrm{P}\mathrm{e}\mathrm{p}\mathrm{t}\mathrm{i}\mathrm{d}\mathrm{e}\:\text{ }\mathrm{c}\mathrm{o}\mathrm{n}\mathrm{c}\mathrm{e}\mathrm{n}\mathrm{t}\mathrm{r}\mathrm{a}\mathrm{t}\mathrm{i}\mathrm{o}\mathrm{n}\:\text{ }\mathrm{o}\mathrm{f}\:\text{ }\mathrm{a}\mathrm{q}\mathrm{u}\mathrm{e}\mathrm{o}\mathrm{u}\mathrm{s}\:\text{ }\mathrm{p}\mathrm{h}\mathrm{a}\mathrm{s}\mathrm{e}\:\text{ }\mathrm{b}\mathrm{e}\mathrm{f}\mathrm{o}\mathrm{r}\mathrm{e}\:\text{ }\mathrm{m}\mathrm{i}\mathrm{x}\mathrm{i}\mathrm{n}\mathrm{g}}\end{aligned}$$

2 $$\begin{aligned}\:\mathrm{P}\mathrm{e}\mathrm{p}\mathrm{t}\mathrm{i}\mathrm{d}\mathrm{e}\:\text{ }\mathrm{l}\mathrm{o}\mathrm{a}\mathrm{d}\mathrm{i}\mathrm{n}\mathrm{g}\:\text{ }(\mathrm{m}\mathrm{g}/\mathrm{g})\:\\=\frac{\mathrm{P}\mathrm{e}\mathrm{p}\mathrm{t}\mathrm{i}\mathrm{d}\mathrm{e}\:\text{ }\mathrm{c}\mathrm{o}\mathrm{n}\mathrm{c}\mathrm{e}\mathrm{n}\mathrm{t}\mathrm{r}\mathrm{a}\mathrm{t}\mathrm{i}\mathrm{o}\mathrm{n}\:\text{ }\mathrm{o}\mathrm{f}\:\text{ }\mathrm{a}\mathrm{q}\mathrm{u}\mathrm{e}\mathrm{o}\mathrm{u}\mathrm{s}\:\text{ }\text{ }\mathrm{p}\mathrm{h}\mathrm{a}\mathrm{s}\mathrm{e}\:\text{ }\mathrm{b}\mathrm{e}\mathrm{f}\mathrm{o}\mathrm{r}\mathrm{e}\:\text{ }\mathrm{m}\mathrm{i}\mathrm{x}\mathrm{i}\mathrm{n}\mathrm{g}\:-\mathrm{P}\mathrm{e}\mathrm{p}\mathrm{t}\mathrm{i}\mathrm{d}\mathrm{e}\:\text{ }\mathrm{c}\mathrm{o}\mathrm{n}\mathrm{c}\mathrm{e}\mathrm{n}\mathrm{t}\mathrm{r}\mathrm{a}\mathrm{t}\mathrm{i}\mathrm{o}\mathrm{n}\:\text{ }\mathrm{o}\mathrm{f}\:\text{ }\mathrm{a}\mathrm{q}\mathrm{u}\mathrm{e}\mathrm{o}\mathrm{u}\mathrm{s}\:\text{ }\mathrm{p}\mathrm{h}\mathrm{a}\mathrm{s}\mathrm{e}\:\text{ }\mathrm{a}\mathrm{f}\mathrm{t}\mathrm{e}\mathrm{r}\:\text{ }\mathrm{c}\mathrm{e}\mathrm{n}\mathrm{t}\mathrm{r}\mathrm{i}\mathrm{f}\mathrm{u}\mathrm{g}\mathrm{a}\mathrm{t}\mathrm{i}\mathrm{o}\mathrm{n}}{\mathrm{S}\mathrm{L}\mathrm{N}\:\text{ }\mathrm{c}\mathrm{o}\mathrm{n}\mathrm{c}\mathrm{e}\mathrm{n}\mathrm{t}\mathrm{r}\mathrm{a}\mathrm{t}\mathrm{i}\mathrm{o}\mathrm{n}\:}\end{aligned}$$

### Measuring in-vitro release kinetics of peptides

In-vitro release kinetics of peptides were studied in a solution of pH 6.5 Tris buffer, which is close to the slightly acidic pH of citrus phloem sap^50^. 10 ml of SLN suspension was added to 40 ml of pH 6.5 Tris buffer, and 1 mL of the solution was withdrawn at predetermined time intervals. The concentration of released peptide was measured using fluorescent spectrometry at excitation and emission wavelengths of 490 nm and 520 nm respectively. Peptide release percentage was calculated using Eq. 3.

3 $$\begin{aligned}\:\mathrm{P}\mathrm{e}\mathrm{p}\mathrm{t}\mathrm{i}\mathrm{d}\mathrm{e}\:\text{ }\mathrm{r}\mathrm{e}\mathrm{l}\mathrm{e}\mathrm{a}\mathrm{s}\mathrm{e}\:\text{ }\mathrm{\%}\:\\=\frac{\mathrm{C}\mathrm{o}\mathrm{n}\mathrm{c}\mathrm{e}\mathrm{n}\mathrm{t}\mathrm{r}\mathrm{a}\mathrm{t}\mathrm{i}\mathrm{o}\mathrm{n}\:\text{ }\mathrm{o}\mathrm{f}\:\text{ }\mathrm{r}\mathrm{e}\mathrm{l}\mathrm{e}\mathrm{a}\mathrm{s}\mathrm{e}\mathrm{d}\:\text{ }\mathrm{p}\mathrm{e}\mathrm{p}\mathrm{t}\mathrm{i}\mathrm{d}\mathrm{e}\:\times\:\:\mathrm{T}\mathrm{o}\mathrm{t}\mathrm{a}\mathrm{l}\:\text{ }\mathrm{v}\mathrm{o}\mathrm{l}\mathrm{u}\mathrm{m}\mathrm{e}\:\text{ }\mathrm{o}\mathrm{f}\:\text{ }\mathrm{s}\mathrm{o}\mathrm{l}\mathrm{u}\mathrm{t}\mathrm{i}\mathrm{o}\mathrm{n}}{\begin{array}{c}\left(\mathrm{P}\mathrm{e}\mathrm{p}\mathrm{t}\mathrm{i}\mathrm{d}\mathrm{e}\:\text{ }\mathrm{c}\mathrm{o}\mathrm{n}\mathrm{c}\mathrm{e}\mathrm{n}\mathrm{t}\mathrm{r}\mathrm{a}\mathrm{t}\mathrm{i}\mathrm{o}\mathrm{n}\:\text{ }\mathrm{o}\mathrm{f}\:\text{ }\mathrm{a}\mathrm{q}\mathrm{u}\mathrm{e}\mathrm{o}\mathrm{u}\mathrm{s}\:\text{ }\mathrm{p}\mathrm{h}\mathrm{a}\mathrm{s}\mathrm{e}\:\text{ }\mathrm{b}\mathrm{e}\mathrm{f}\mathrm{o}\mathrm{r}\mathrm{e}\:\text{ }\mathrm{m}\mathrm{i}\mathrm{x}\mathrm{i}\mathrm{n}\mathrm{g}\:-\:\mathrm{P}\mathrm{e}\mathrm{p}\mathrm{t}\mathrm{i}\mathrm{d}\mathrm{e}\:\text{ }\mathrm{c}\mathrm{o}\mathrm{n}\mathrm{c}\mathrm{e}\mathrm{n}\mathrm{t}\mathrm{r}\mathrm{a}\mathrm{t}\mathrm{i}\mathrm{o}\mathrm{n}\:\text{ }\mathrm{o}\mathrm{f}\:\text{ }\mathrm{a}\mathrm{q}\mathrm{u}\mathrm{e}\mathrm{o}\mathrm{u}\mathrm{s}\:\text{ }\mathrm{p}\mathrm{h}\mathrm{a}\mathrm{s}\mathrm{e}\:\text{ }\mathrm{a}\mathrm{f}\mathrm{t}\mathrm{e}\mathrm{r}\:\text{ }\mathrm{c}\mathrm{e}\mathrm{n}\mathrm{t}\mathrm{r}\mathrm{i}\mathrm{f}\mathrm{u}\mathrm{g}\mathrm{a}\mathrm{t}\mathrm{i}\mathrm{o}\mathrm{n}\right)\\\:\times\:\:\mathrm{V}\mathrm{o}\mathrm{l}\mathrm{m}\mathrm{e}\:\text{ }\mathrm{o}\mathrm{f}\:\text{ }\mathrm{t}\mathrm{h}\mathrm{e}\text{ }\mathrm{S}\mathrm{L}\mathrm{N}\mathrm{s}\text{ }\mathrm{a}\mathrm{d}\mathrm{d}\mathrm{e}\mathrm{d}\end{array}}\end{aligned}$$

### Transmission electron microscopy (TEM) imaging of SLNs

Cryo-TEM samples were prepared by applying 4 µL of SNPs (20 mg/mL total lipid) to standard electron microscopy grids coated with a perforated carbon film (Quantifoil Micro Tools GmbH). Excess liquid was removed by blotting, and the grids were plunge-frozen in liquid ethane using a Vitrobot Mark IV system (FEI, Hillsboro, OR) operated at RT and 100% humidity with a blot force of −4. Frozen grids were imaged under cryogenic conditions (~ 88 K) using a Titan Krios transmission electron microscope (Thermo Fisher Scientific) equipped with a Gatan K3 direct electron detector. Images were acquired at a nominal magnification of 53,000× under low-dose conditions with a defocus range of − 1 to − 3 μm to enhance contrast. Samples were loaded using an Autoloader, and all experiments were conducted at the Laboratory of Biomolecular Structure and Dynamics (LBSD-CryoEM) at Texas A&M University, which is partly supported by CPRIT Facility Support Grant (RP25049).

### Fluorescence imaging of SLNs

Fluorescence images of particles were taken using a Leica SP8 (Leica Microsystems, Wetzlar, Germany) confocal microscope equipped with Hybrid Detectors and the FALCON fluorescence lifetime imaging (FLIM) module. The protein was tagged with 5-carboxyfluorescein (FAM), and the lipid was tagged with Nile Red. A final Nile Red concentration of 0.5 µg/mL was used, which is within the generally used range^51^. In order to reduce photo bleaching of fluorescein, 2% (w/w) of propyl gallate was used as an anti-fade reagent, and the pH was maintained at 8.0 using pH 8 Tris buffer^52^. The residual protein not incorporated into SLNs was removed by filtration through a membrane with a 3.5 kDa cutoff. Filtered samples were immobilized in 2% agarose gel and were imaged using a 40×/1.1 water immersion objective. Excitation wavelengths of 488 nm and 577 nm, and emission wavelength ranges of 499–565 nm and 587–659 nm were used for Fluorescein and Nile Red, respectively^53,54^, in a line-sequential mode to minimize spectral cross-talk between the two image channels. The presence and identity of each fluorescent dye were further verified using FLIM.

### Determining the penetration of SLNs into the vascular system

Penetration of peptide-loaded SLNs into the vascular system was determined by fluorescence imaging of vascular tissues following the foliar application of SLNs. Meyer Lemon (*Citrus meyeri*) plants in 1-gal containers were used for this experiment. The plants were kept under full-spectrum grow lights and watered every two days. Two milliliters of SLNs were applied to the surface of each leaf using a pipette, and were kept for a predetermined time period. The treated leaves were separated from the tree, rinsed thoroughly with deionized water to remove peptides remaining on the surface, and both longitudinal and cross sections of the petiole were cut using a razor blade. Sections were placed in a drop of water in a cover glass-bottom imaging chamber and analyzed using a Leica SP8 confocal microscope with a 20x/0.75 water immersion objective using the same excitation and emission settings as described above, again in line sequential mode of acquisition. An additional image channel of chlorophyll fluorescence with a detection range of 675–700 nm^55^ was captured simultaneously with the Nile Red channel to image the chloroplasts and determine if there was any bleed-through of the chlorophyll fluorescence signal into the Fluorescein or Nile Red channels. Brightfield images were captured simultaneously with the Fluorescein signal for the initial focusing and to identify the vascular tissues in the sample. Same protocol was used for the leaves treated with aqueous peptide.

### Analysis of fluorescence images

Acquisition of fluorescence images for each color channel and analysis of FLIM data were done using Leica LASX software v.3.5.7 with the Phasor FLIM analysis module. Mean fluorescence, particle density, and particle area of acquired images were measured using Fiji ImageJ after setting the threshold using the default method and the LAB color space. Only the particles above 8 pixel size diameter (corresponding to approximately 400 µM) were considered for particle density and area analysis. Corrected mean fluorescence and particle density were calculated using Eqs. 4 and 5. All fluorescence parameters were calculated based on multiple images provided in the Supplementary data.

4 $$\rm{Corrected \text{ } mean \text{ }fluorescence = Mean \text{ }fluorescence \text{ }of \text{ }the \text{ }sample-Mean \text{ }fluorescence \text{ }of \text{ }the \text{ }control}$$

5 $$\:\mathrm{C}\mathrm{o}\mathrm{r}\mathrm{r}\mathrm{e}\mathrm{c}\mathrm{t}\mathrm{e}\mathrm{d}\:\text{ }\mathrm{p}\mathrm{a}\mathrm{r}\mathrm{t}\mathrm{i}\mathrm{c}\mathrm{l}\mathrm{e}\text{ }\:\mathrm{d}\mathrm{e}\mathrm{n}\mathrm{s}\mathrm{i}\mathrm{t}\mathrm{y}\:=\frac{\mathrm{P}\mathrm{a}\mathrm{r}\mathrm{t}\mathrm{i}\mathrm{c}\mathrm{l}\mathrm{e}\text{ }\:\mathrm{c}\mathrm{o}\mathrm{u}\mathrm{n}\mathrm{t}\text{ }\:\mathrm{o}\mathrm{f}\text{ }\:\mathrm{s}\mathrm{a}\mathrm{m}\mathrm{p}\mathrm{l}\mathrm{e}}{\mathrm{A}\mathrm{r}\mathrm{e}\mathrm{a}\text{ }\:\mathrm{o}\mathrm{f}\text{ }\:\mathrm{s}\mathrm{a}\mathrm{m}\mathrm{p}\mathrm{l}\mathrm{e}}-\:\frac{\mathrm{P}\mathrm{a}\mathrm{r}\mathrm{t}\mathrm{i}\mathrm{c}\mathrm{l}\mathrm{e}\text{ }\:\mathrm{c}\mathrm{o}\mathrm{u}\mathrm{n}\mathrm{t}\text{ }\:\mathrm{o}\mathrm{f}\text{ }\:\mathrm{c}\mathrm{o}\mathrm{n}\mathrm{t}\mathrm{r}\mathrm{o}\mathrm{l}}{\mathrm{A}\mathrm{r}\mathrm{e}\mathrm{a}\text{ }\:\mathrm{o}\mathrm{f}\text{ }\:\mathrm{c}\mathrm{o}\mathrm{n}\mathrm{t}\mathrm{r}\mathrm{o}\mathrm{l}}$$

Colocalization between red and green channels was quantified using BIOP JACOP Plugin in Fiji. Pearson’s correlation coefficient and Manders’ coefficients were calculated after Costes automatic thresholding. Statistical significance was assessed using Costes’ randomization test with 100 shuffles.

### MALDI-MS analysis

Matrix-Assisted Laser Desorption/Ionization – Time of Flight (MALDI-TOF) mass spectrometry of samples was conducted using a Bruker Ultraflextreme MALDI-TOF Mass Spectrometer. Protein extraction of treated leaves was done using the reported protocols^56^. The leaves were rinsed thoroughly and ground in liquid nitrogen. 1 mL of extraction buffer containing 90% acetone, 10% trichloroacetic acid, and 0.007% β-mercaptoethanol was added to the ground tissues and vortexed. Following 1 h incubation at −20 °C, the samples were centrifuged for 15 min at 6000 rpm, and the pellet was washed three times with acetone containing 2 mM Ethylenediaminetetraacetic acid (EDTA). Washed pellets were vacuum dried and resuspended in 250 µL of solubilization buffer containing Urea, Thiourea, and 3-((3-Cholamidopropyl)dimethylammonio)−1-propanesulfonate (CHAPS). The resuspended solutions were centrifuged at 6000 rpm for 15 min, and the 40 µL of supernatant was mixed with 5 µL of 1% trifluoroacetic acid (TFA) and 5 µL of 100 µg/mL Melittin solution as an internal standard. These samples were concentrated using Thermo Scientific C18 ziptip filters and eluted with 2µL of α-Cyano-4-hydroxycinnamic acid (CHCA) matrix to the MALDI target plate. The mass spectra were obtained with a 90% laser.

Acquisition of spectra and detection of peaks was done using Flex Control/Flex Analysis^®^ software, and peak areas were calculated using Microsoft Excel using the Trapezoidal method. Relative peak intensities and relative peak areas for Peptide 57,020 were calculated using Eqs. 6 and 7.

6 $$\:\mathrm{R}\mathrm{e}\mathrm{l}\mathrm{a}\mathrm{t}\mathrm{i}\mathrm{v}\mathrm{e}\text{ }\:\mathrm{p}\mathrm{e}\mathrm{a}\mathrm{k}\text{ }\:\mathrm{i}\mathrm{n}\mathrm{t}\mathrm{e}\mathrm{n}\mathrm{s}\mathrm{i}\mathrm{t}\mathrm{y}=\:\frac{\mathrm{P}\mathrm{e}\mathrm{a}\mathrm{k}\text{ }\:\mathrm{i}\mathrm{n}\mathrm{t}\mathrm{e}\mathrm{n}\mathrm{s}\mathrm{i}\mathrm{t}\mathrm{y}\text{ }\:\mathrm{o}\mathrm{f}\text{ }\:\mathrm{P}\mathrm{e}\mathrm{p}\mathrm{t}\mathrm{i}\mathrm{d}\mathrm{e}\text{ }\:57020}{\mathrm{P}\mathrm{e}\mathrm{a}\mathrm{k}\text{ }\:\mathrm{i}\mathrm{n}\mathrm{t}\mathrm{e}\mathrm{n}\mathrm{s}\mathrm{i}\mathrm{t}\mathrm{y}\text{ }\:\mathrm{o}\mathrm{f}\text{ }\:\mathrm{M}\mathrm{e}\mathrm{l}\mathrm{i}\mathrm{t}\mathrm{t}\mathrm{i}\mathrm{n}}$$

7 $$\:\mathrm{R}\mathrm{e}\mathrm{l}\mathrm{a}\mathrm{t}\mathrm{i}\mathrm{v}\mathrm{e}\text{ }\:\mathrm{p}\mathrm{e}\mathrm{a}\mathrm{k}\text{ }\:\mathrm{a}\mathrm{r}\mathrm{e}\mathrm{a}=\:\frac{\mathrm{P}\mathrm{e}\mathrm{a}\mathrm{k}\text{ }\:\mathrm{a}\mathrm{r}\mathrm{e}\mathrm{a}\text{ }\:\mathrm{o}\mathrm{f}\text{ }\:\mathrm{P}\mathrm{e}\mathrm{p}\mathrm{t}\mathrm{i}\mathrm{d}\mathrm{e}\text{ }\:57020}{\mathrm{P}\mathrm{e}\mathrm{a}\mathrm{k}\text{ }\:\mathrm{a}\mathrm{r}\mathrm{e}\mathrm{a}\text{ }\:\mathrm{o}\mathrm{f}\text{ }\:\mathrm{M}\mathrm{e}\mathrm{l}\mathrm{i}\mathrm{t}\mathrm{t}\mathrm{i}\mathrm{n}}$$

### Measurement of Chlorophyll content

Chlorophyll content of treated leaves was monitored to detect phytotoxicity. Chlorophyll content of leaves was measured daily at the same time for 10 days using the GYJ-C non-invasive Chlorophyll analyzer. Measurements were taken from three leaves for each treatment, at two locations on each leaf.

### Statistical analysis

Statistical analysis for this study was conducted using JMP Pro 17. A second-degree full factorial model was used to analyze the impact of Tween 80 and poly(DADMAC) concentrations on particle size and zeta potential, and the impact of variables was determined using effect tests. All experiments were conducted in triplicate.

Statistical analysis of fluorescent parameters and MALDI-MS peak data of SLN-treated and aqueous peptide-treated samples was done using Welch’s t-test for unequal variances, and analysis of leaf chlorophyll content was done using Tukey’s Honestly Significant Difference (HSD) approach.

## Results and discussion

### Variation of particle size and zeta potential with nanoparticle composition

**Fig. 1:**
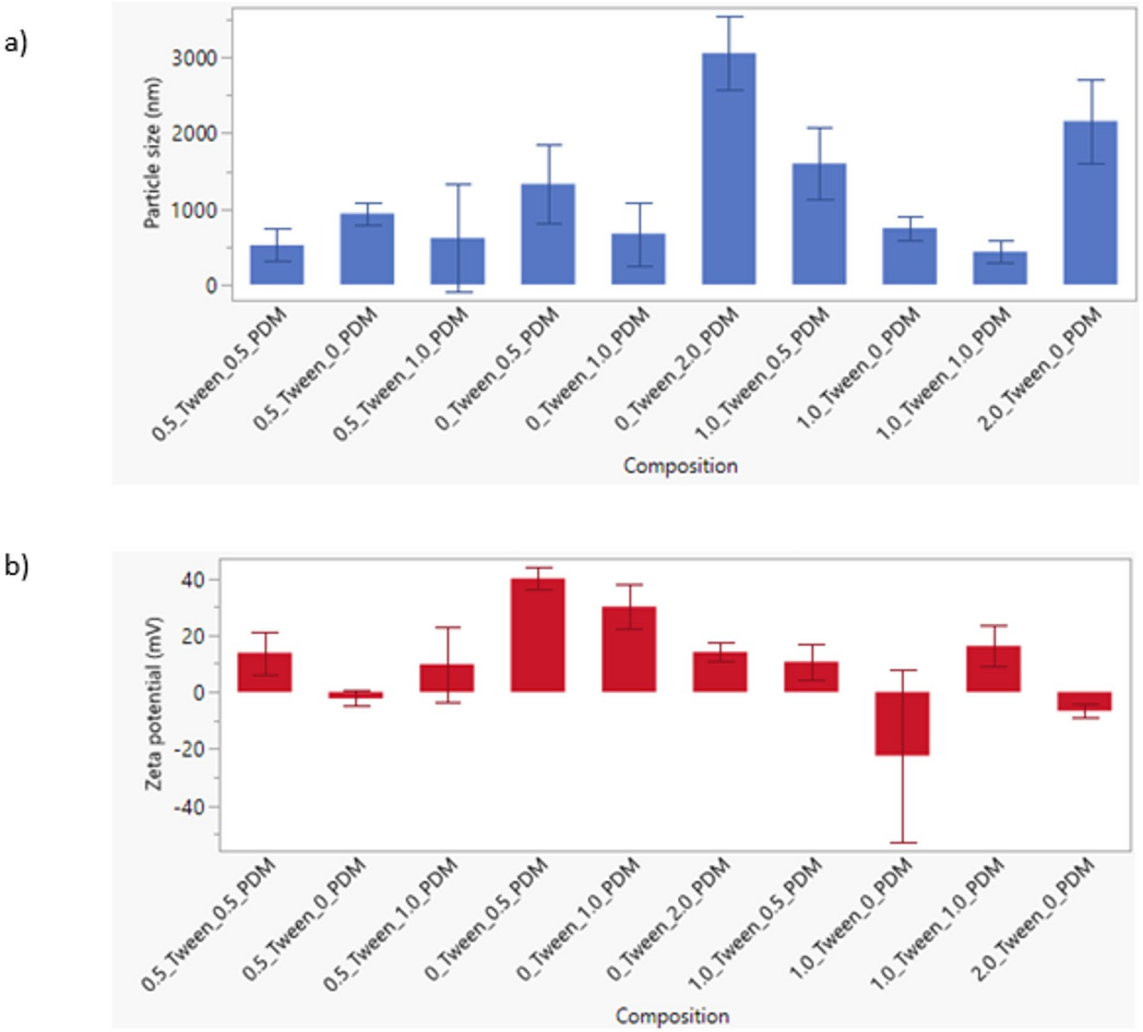
Variation of (**a**) particle size and (**b**) zeta potential of SLNs with composition. Compositions are labeled by Tween 80 composition followed by poly(DADMAC) concentration. As an example, SLNs labeled as 0.5_Tween_0.5_PDM contains 0.5% Tween 80 and 0.5% poly(DADMAC).

Particle size and zeta potential are important parameters of nanoparticles, which affect particle stability and cellular uptake. Therefore, maintaining particle size and zeta potential at desired levels is essential when using nanoparticles for drug delivery applications^57,58^. Particle size and zeta potential at different nanoparticle compositions are given in Fig. 1.

As shown in Fig. 1, the smallest average particle size of 435.3 nm was obtained at 1% Tween 80 and 1% poly(DADMAC) composition, while 0.5% Tween 80 and 0.5% poly(DADMAC) composition gave the second smallest particle size. In general, compositions containing both poly(DADMAC) and Tween 80 showed smaller particle sizes than compositions with only one of them, suggesting a possible synergistic effect of the two compounds. It was also observed that increasing Tween 80 or poly(DADMAC) content from 1% to 2% led to a large increase in particle size, which could be a result of particle aggregation under too high surfactant concentration^59,60^.

The zeta potential of nanoparticles showed a strong relationship with the poly(DADMAC) concentration. It can be clearly observed that all compositions containing poly(DADMAC) showed a significant positive charge above 10 mV. This is expected as poly(DADMAC) is a cationic polymer with a strong positive charge^38,61^. Although Tween 80 is a nonionic surfactant, a small negative charge could be observed in compositions containing only Tween 80, which could be attributed to the adsorption of ionic species such as OH^−^ ions from water and free fatty acids from lipids into the particle surfaces^33,62^. Particles that contained both Tween 80 and poly(DADMAC) showed positive charges, since the positive charge of poly(DADMAC) is much stronger than the effects of Tween 80.

The impact of Tween 80 and poly(DADMAC) concentrations on particle size and zeta potential was further evaluated using statistical analysis using a second-degree full factorial model. The detailed results of this statistical analysis are presented in supplementary Table S2. Particle size strongly depended on both Tween 80 and poly(DADMAC) concentrations, with p-values of 0.0012 and < 0.0001, respectively. The interaction between Tween 80 and poly(DADMAC) concentration had a p-value of 0.0578, which was not significant under a 95% confidence level, but was significant at a 90% confidence level. Thus, both Tween 80 and poly(DADMAC) concentrations, and their interactions, show a significant impact on particle size at 90% confidence.

In comparison, only poly(DADMAC) concentration showed a significant impact on zeta potential, with a p-value of 0.0402. Neither Tween 80 concentration nor the interaction between the two compounds showed any significant impact on zeta potential, even at a 90% confidence level. This result is consistent with the strong positive charge in poly(DADMAC).

For drug delivery purposes, smaller particle sizes and higher positive charges are more desirable, since small particle sizes have been reported to result in higher cellular uptake^22–24,63–65^, and a strong positive charge can help the permeation of the negatively charged cell surface^32,33,38,66–68^. Since 0.5% Tween 80 + 0.5% poly(DADMAC), and 1.0% Tween 80 + 1.0% poly(DADMAC) compositions resulted in the smallest particle size and also showed a significant positive charge, those compositions were used for subsequent experiments.

### Peptide encapsulation into nanoparticles

Peptide encapsulation into nanoparticles was verified by calculating encapsulating efficiency and peptide loading, and fluorescence imaging of SLNs. Encapsulation efficiency and peptide loading of the two screened SLN compositions are given in Table 1.

**Table 1:** Encapsulation efficiency and peptide loading of SLNs determined using fluorescence spectrometry.

SLN composition	Encapsulation efficiency (%)	Peptide loading (mg/g)
0.5% Tween 80 + 0.5% poly(DADMAC)	94.8 ± 0.8	0.836 ± 0.073
1.0% Tween 80 + 1.0% poly(DADMAC)	84.8 ± 16.7	0.689 ± 0.119

As observed in Table 1, SLNs with 0.5% Tween 80 and 0.5% poly(DADMAC) showed the higher encapsulation efficiency of 94.8%, which is very good compared with the encapsulation efficiencies of peptides in SLNs reported by other researchers^69–71^. The encapsulation efficiency of SLNs with 1.0% Tween 80 and 1.0% poly(DADMAC) was slightly lower at 84.8%. This behavior can be attributed to the positive charge of the peptide caused by the four Arginine residues, which can cause repulsion between peptide and poly(DADMAC) molecules at higher poly(DADMAC) concentrations^72^. Since 0.5% Tween 80 and 0.5% poly(DADMAC) composition showed better encapsulation efficiency, subsequent experiments were conducted using SLNs with this composition.

### In-vitro peptide release kinetics

**Fig. 2:**
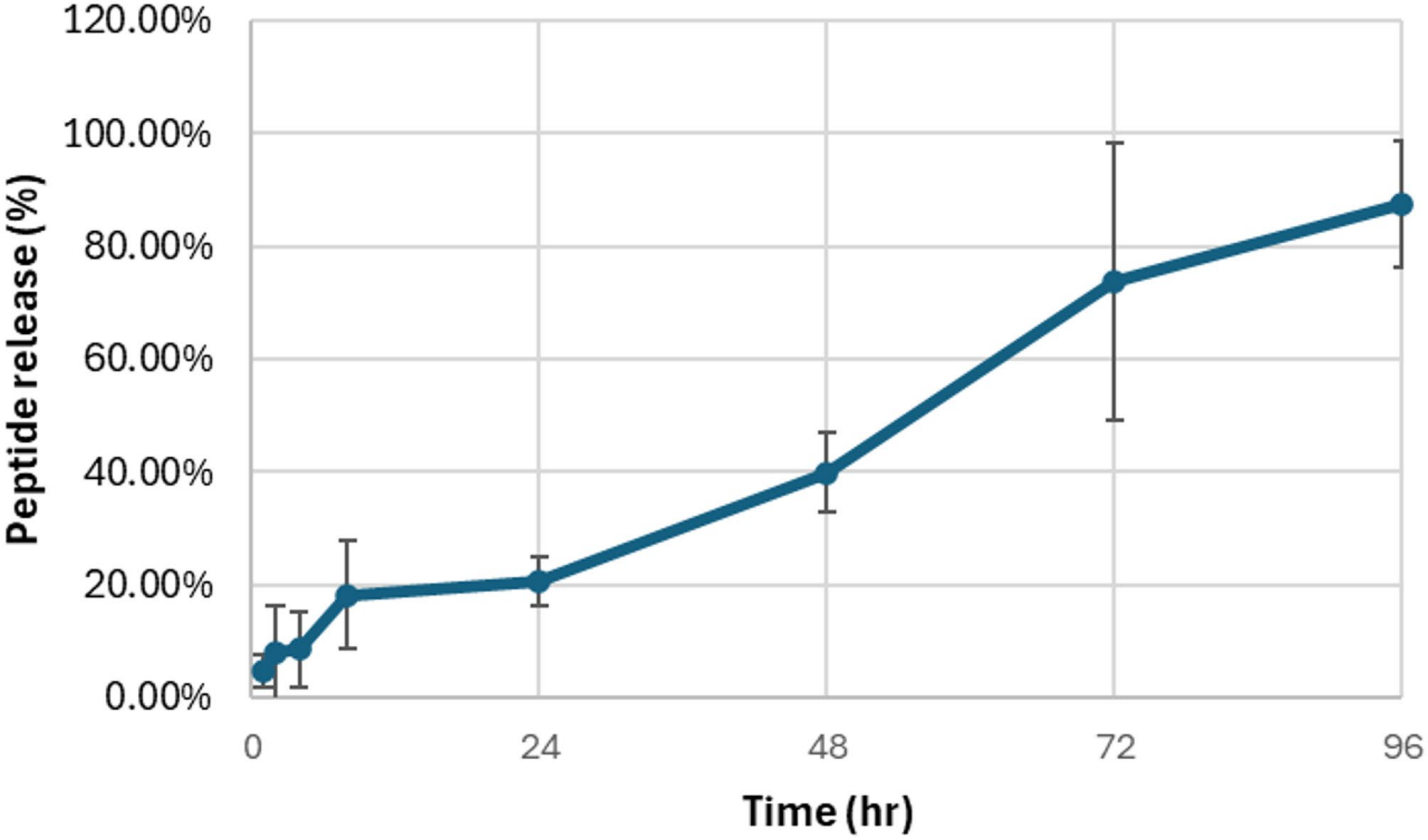
In-vitro release of peptides from SLNs over time. The error bars represent ± standard deviation. The peptide release curve steady peptide release which follows zeroth order kinetics.

In-vitro peptide release curve (Fig. 2) demonstrated consistent peptide release over an extended time period. There was an initial burst release, with around 20% of the peptide being released within the first 8 h, followed by consistent release of peptide up to 72 h. A decrease in release rate was observed during the last 24 h, which is expected due to the depletion of encapsulated peptide and the reduction in concentration gradient. The peptide release predominantly followed zeroth order drug release kinetics (R^2^ = 0.98). This steady drug release profile makes the SLNs highly desirable for peptide delivery, as this will enable maintaining desired concentrations over extended time periods without frequent redosing^73^.

### TEM imaging of SLNs

**Fig. 3:**
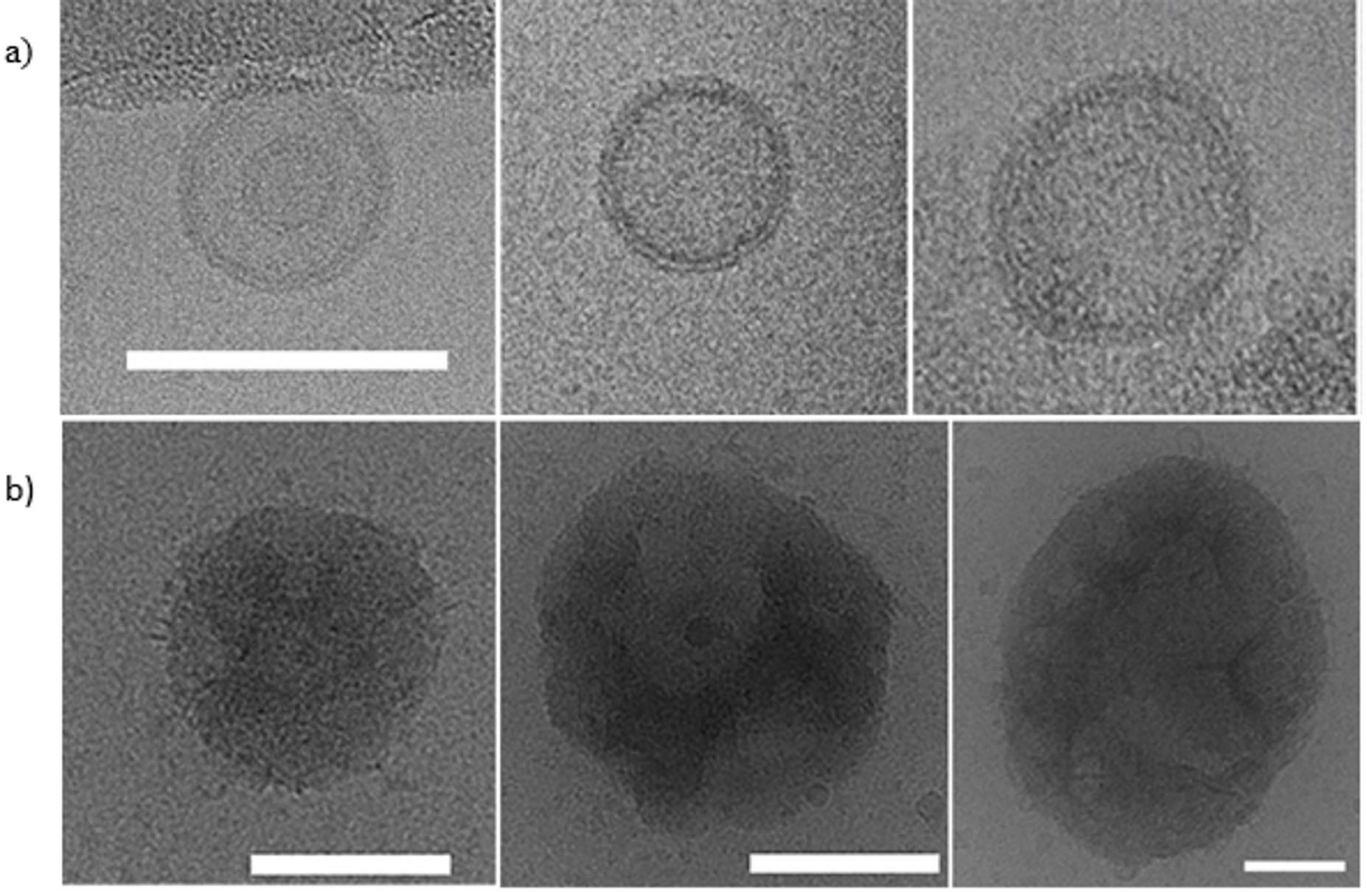
TEM images of (**a**) unloaded and (**b**) peptide-loaded SLNs. The darker cores in peptide-loaded SLN indicate peptide encapsulation. Both unloaded and peptide-loaded SLNs display spherical geometry, with peptide-loaded SLNs displaying some surface irregularities. Presence of poly(DADMAC) is shown as a peripheral corona. Scale bars indicate 100 nm.

TEM images of the SLNs were acquired to determine the structural morphology and verify encapsulation. Unloaded SLNs (Fig. 3a) displayed a spherical morphology, with well-defined boundaries and a uniform density in the core. The darker corona at the edges suggests that poly(DADMAC) is present as a thin layer surrounding the lipid cores. Peptide-loaded SLNs (Fig. 3b) retain the overall spherical morphology, but display rougher edges than the unloaded SLNs. Peptide-loaded SLNs exhibited darker cores indicating that the peptides are encapsulated within the lipid core^74^.

### Fluorescence imaging of SLNs

**Fig. 4:**
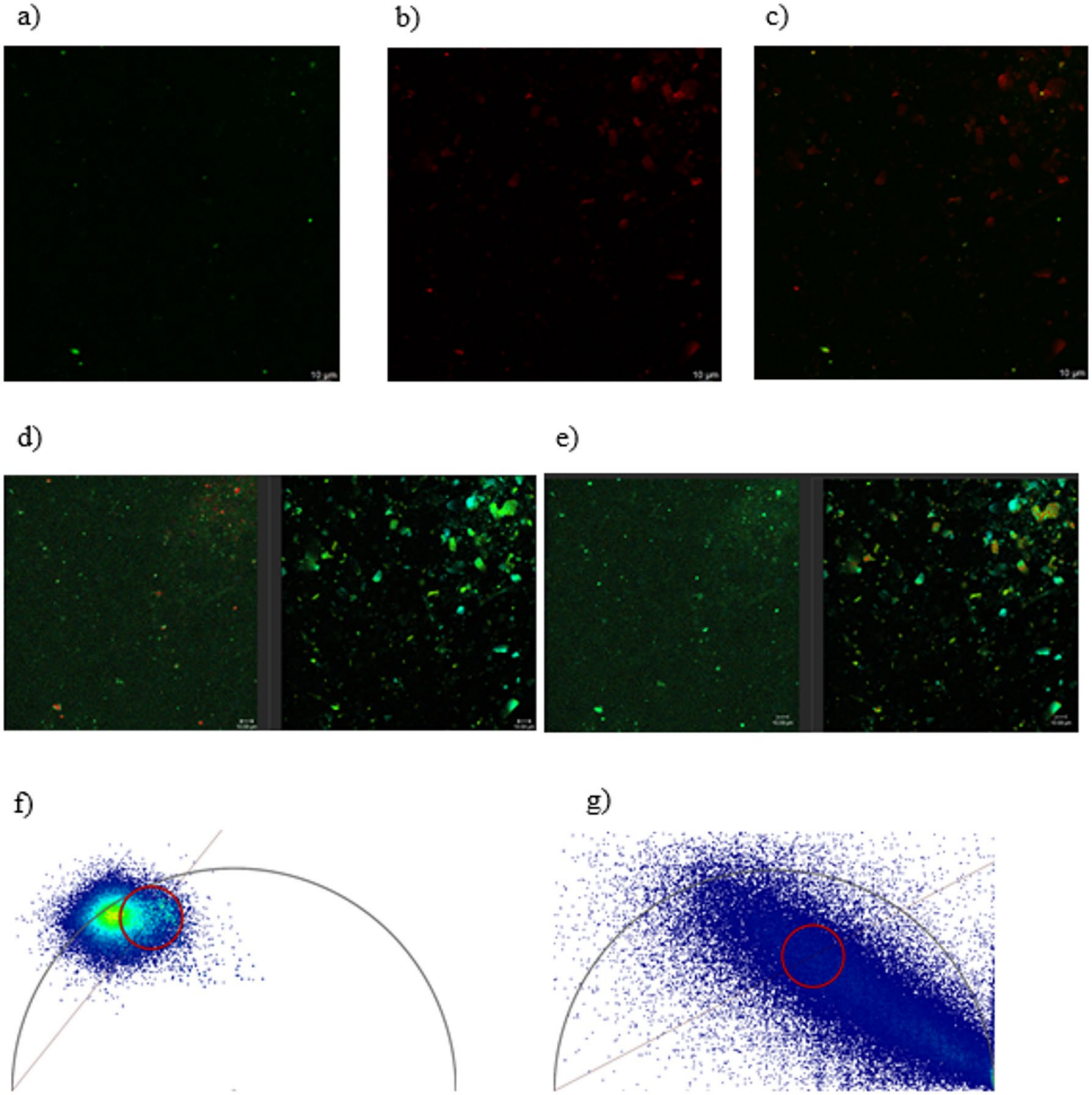
Fluorescence images of immobilized SLNs in (**a**) Fluorescein channel (499–565 nm), (**b**) Nile Red channel (587–659 nm) and (**c**) overlay of Fluorescein and Nile Red channels; (**d**) regions with fluorescence lifetime close to 2.5 ns (shown in red) in Nile Red (left) and Fluorescein (right) channels; (**e**) regions with fluorescence lifetime close to 1.06 ns (shown in red) in Nile Red (left) and Fluorescein (right) channels; and Phasor plots corresponding to fluorescence life times of (**f**) 2.5 ns and (**g**) 1.06 ns. These results suggest no significant interference between Fluorescein and Nile Red channels. Combined wavelength and lifetime analysis confirms successful peptide encapsulation within SLNs, and demonstrates that Fluorescein and Nile Red signals remain resolvable without significant spectral or temporal interference.

Fluorescence imaging of SLNs was used to verify encapsulation of peptides within the lipids, using both wavelength and fluorescence lifetime to distinguish between the two fluorophores (Fig. 4). As seen in Fig. 4a-c, most of the bright spots in the Fluorescein channel had corresponding bright spots in the Nile Red channel, suggesting that the regions with high concentrations of Fluorescein also had a high concentration of Nile Red, which is indicative of successful encapsulation of the peptide within the lipids. This observation was supported by colocalization analysis, which showed an R_obs (observed correlation coefficient) of 0.264 compared to an R_and (random correlation coefficient) of 0.083, with a Jacob’s p-value of 1.0. R_obs quantifies the spatial overlap between the two fluorescent signals, while R_and represents the expected random overlap. Jacob p-value was used to measure the probability of R_obs being significantly higher than R_and^75^. The Jacob p-value of 1.0 indicates that the overlap between the two signals was statistically significant and is not a result of random overlap. Further quantification using Manders’ coefficients showed that 16.4% of the fluorescein signal overlapped with the Nile Red signal, while 34.5% of the Nile Red signal colocalized with fluorescein, consistent with localized peptide encapsulation.

The composition of the bright spots was further analyzed based on fluorescence lifetime. As observed in Fig. 4d and e, the bright spots in the Fluorescein and Nile Red channels had fluorescence lifetimes close to 2.5 ns and 1.06 ns, respectively, suggesting that the fluorescence in these two channels is caused by two different components. The Nile Red channel had a significantly shorter lifetime than the Fluorescein channel, which agrees with the literature, which reports that Nile Red generally has a shorter lifetime than Fluorescein^76,77^. While fluorescence lifetimes of the bright spots in Fluorescein and Nile Red channels were shorter than the reported lifetimes for Fluorescein and Nile Red^76,77^, it is a potential result of aggregation-caused quenching^78,79^. The shorter lifetime of Nile Red can also be attributed to the polar nature of SLNs due to the high charge density of poly(DADMAC), as Nile Red is reported to show shorter lifetimes under higher polarity^77^.

Phasor analysis was used to visualize and confirm the separation of these lifetime populations without the need for curve fitting. In the phasor plots (Fig. 4f and g), pixels from the Fluorescein channel cluster near the position corresponding to ~ 2.5 ns, whereas pixels from the Nile Red channel cluster near the ~ 1.06 ns position, clearly indicating that the two channels can be distinguished based solely on lifetime differences. The broader spread of Nile Red points in the phasor plot likely reflects heterogeneity in its local microenvironment within the SLNs, consistent with its known sensitivity to polarity near charged interfaces^80^.

### Permeation of SLNs into the vascular system

**Fig. 5:**
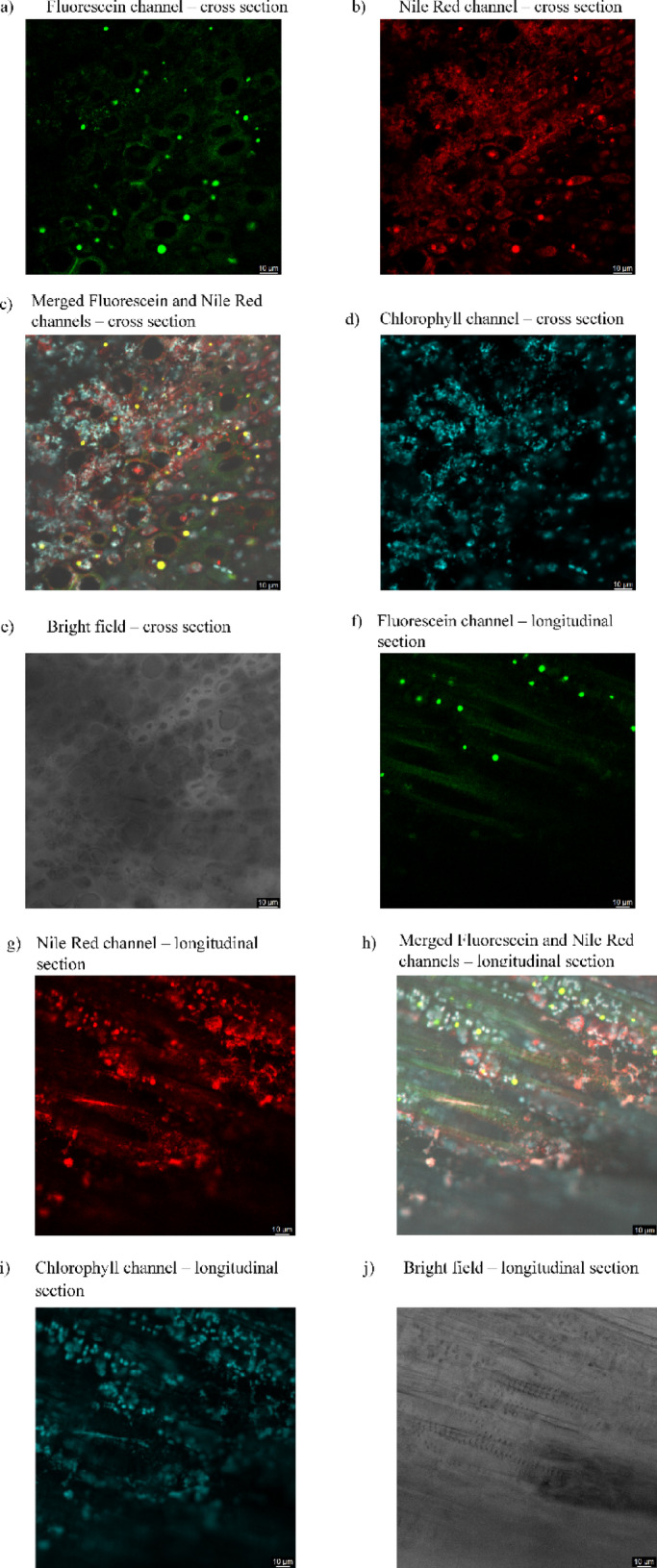
Fluorescence images of a cross section of the petiole of leaves treated with SLNs in (**a**) Fluorescein channel (499–565 nm), (**b**) Nile Red channel (587–659 nm), (**c**) merged Fluorescein and Nile Red channels, (**d**) chlorophyll channel (675–700 nm), and (**e**) bright field image of the cross section; and fluorescence images of a longitudinal section of the petiole in (**f**) Fluorescein channel (499–565 nm), (**g**) Nile Red channel (587–659 nm), (**h**) merged Fluorescein and Nile Red channels, (**i**) chlorophyll channel (675–700 nm), and (**j**) bright field image of the longitudinal section. Samples were taken 24 h after foliar application of the SLNs. Multiple fluorescence spots can be observed in the SLN-treated sample in both Fluorescein and Nile Red channels. This confirms that peptides have successfully permeated into vascular tissues in SLN-treated samples. The image used in Fig. 5a is reused in Fig. 6a and Fig 7b, and the image used in Fig. 5f is reused in Fig. 6d in different comparisons.

In order to determine if the SLNs can penetrate the leaves and enter the plant vascular system, samples of the petiole were analyzed using fluorescence microscopy 24 h after foliar application of the SLNs. Fluorescence images of a cross-section and a longitudinal section of the petiole are depicted in Fig. 5.

As shown in Fig. 5, a number of bright spots can be observed in the Fluorescein channel of both cross-section and longitudinal sections, which suggests the presence of fluorescein inside the tissues. Some of these bright spots have a diameter close to the measured nanoparticle size, while the larger spots have a diameter in the 1–2 µM range, which could be due to the aggregation of nanoparticles. Nile Red and Chlorophyll channels of the treated sample were also analyzed to determine if there was an interference due to the autofluorescence of plant tissues. It could be observed that while the Chlorophyll channel had some interference in the Nile Red channel, it did not cause any noticeable interference in the Fluorescein channel. The bright spots in the Fluorescein channel could also be observed in the Nile Red channel, distinguishable from the chloroplasts. The presence of bright spots in both Fluorescein and Nile Red channels, and not in the Chlorophyll channel, indicates that those spots represent the SLNs. This was further verified using colocalization analysis, which revealed an observed correlation coefficient (R_obs) of 0.391, and a Jacob’s p-value of 1.0, indicating statistically significant colocalization between Nile Red and Fluorescein channels. Thresholded Manders’ coefficients indicated that 45.7% of the Fluorescein signal overlapped with Nile Red signal, while 34.4% of the Nile Red signal colocalized with Fluorescein signal. The tissue samples demonstrated higher colocalization compared to immobilized SLNs, indicating that peptide signal within vascular tissues is largely associated with SLNs.

**Fig. 6:**
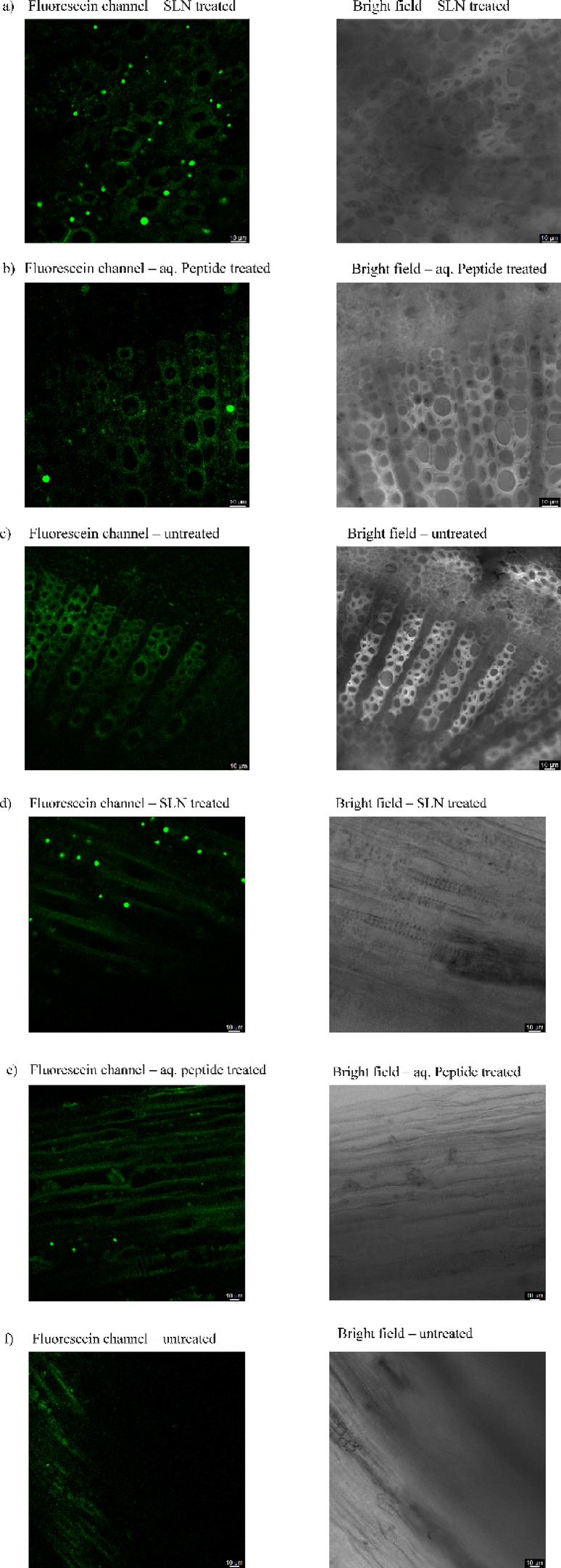
Fluorescence images in Fluorescein channel (499–565 nm) and bright field images of cross sections of the petiole of (**a**) sample treated with SLNs containing 40 mg/L concentration of peptide 57,020, (**b**) sample treated with 40 mg/L concentration of aqueous peptide 57,020, (**c**) untreated sample; images of longitudinal sections of the petiole of (**d**) sample treated with SLNs containing 40 mg/L concentration of peptide 57,020, (**e**) sample treated with 40 mg/L concentration of aqueous peptide 57,020, (**f**) untreated sample. **a**,** b** and **c** depict cross-sectional images and **d**,** e** and **f** depict longitudinal images. In each sub figure, fluorescein channel is shown on the left, and the bright field channel is shown on the right. Samples treated with SLNs showed greater fluorescence than samples treated with aqueous peptides. Fig. 6a and Fig. 6d have been previously used in Fig. 5a and 5f respectively for a different comparison.

A comparison of fluorescence images of samples treated with SLNs and aqueous peptides was used to determine if SLNs can facilitate the delivery of the peptides into vascular tissues (Fig. 6). As observed in Fig. 6, the number of bright spots in the Fluorescein channel of the sample treated with SLNs was much higher than that of the sample treated with aqueous peptide solution, suggesting that encapsulation of the peptide within SLNs can enhance the delivery of peptides into the vascular system. A smaller number of bright spots with low intensity could also be observed in the sample treated with aqueous peptides, suggesting that a small amount of peptides could directly enter the vascular tissues via foliar application, which can be significantly enhanced via SLNs.

Analysis of fluorescence parameters was conducted to further evaluate the imaging results. Corrected mean fluorescence of SLN-treated and aqueous peptide-treated samples after 24 h of treatment are given in Table 2. The images used for fluorescence parameter calculation are included in Supplementary Figure S1.

**Table 2:** Fluorescence parameters of SLN-treated and aqueous peptide-treated samples after 24 h of treatment.

Parameter	SLN-treated samples	Aqueous peptide-treated samples	Untreated samples (control)
Mean fluorescence	5.291 ± 0.723	4.850 ± 1.326	3.695 ± 0.824
Corrected mean fluorescence	1.596	1.155	N/A

According to Table 2, SLN-treated samples showed a higher corrected mean fluorescence than aqueous peptide-treated samples. However, the difference in mean fluorescence was not significant (*p* = 0.4959), likely due to the high levels of background noise from plant tissues. Therefore, the fluorescent intensity data is inadequate to provide quantitative evidence of peptide delivery and should only be considered as qualitative data. MALDI-MS was conducted as a quantitative technique to assess the presence of peptides within the plant tissues.

**Fig. 7:**
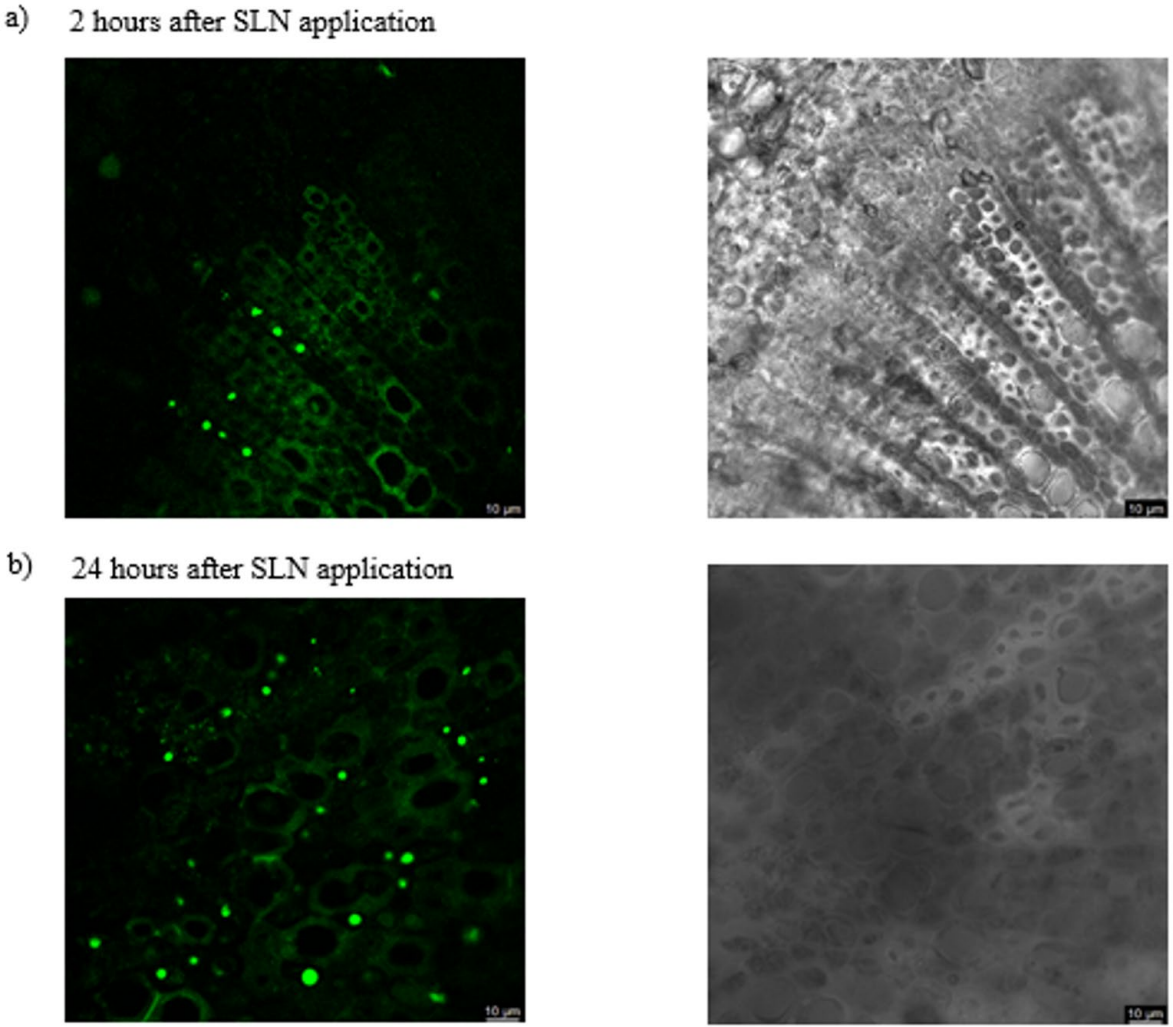
Fluorescence images of the Fluorescein channel (499–565 nm) and bright field images of the cross sections of the petiole, (**a**) 2 h and (**b**) 24 h after SLN application. Fluorescein channel is shown on the left, and the bright field channel is shown on the right. Greater fluorescence could be observed after 24 h, suggesting continuous migration of peptides from the surface to the vascular tissues. Fig. 7b has been previously used in Fig. 5a and Fig. 6a for different comparisons.

To determine the impact of time on the movement of peptides, we analyzed the fluorescence images of inner vascular tissues at different time periods after application of SLNs (Fig. 7). As shown in Fig. 7, the sample after 24 h of treatment shows more fluorescence compared to the sample after 2 h. This observation suggests continuous migration of the peptides from the surface of the leaves towards the vascular tissues.

### Movement of SLNs through the vascular system

**Fig. 8:**
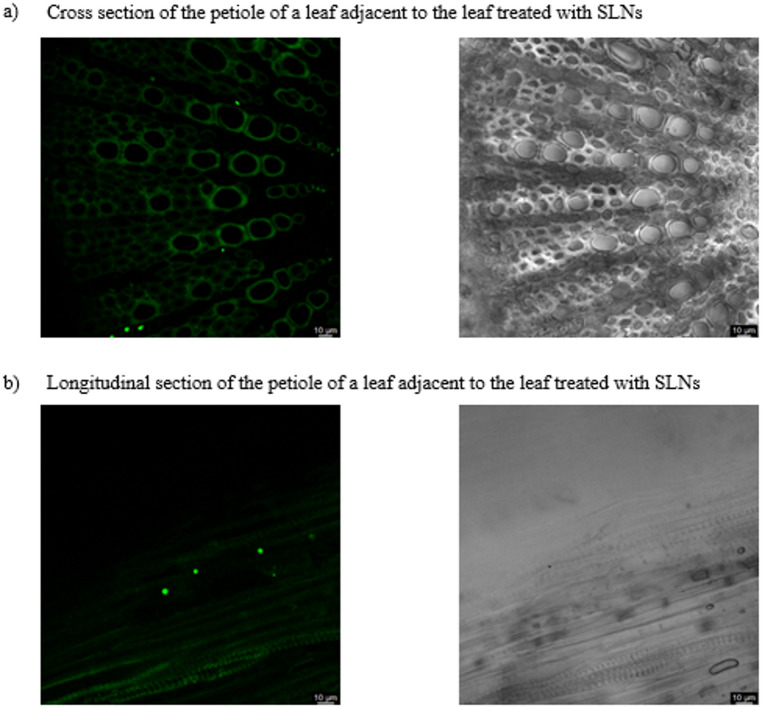
Fluorescence images of the Fluorescein channel (499–565 nm) and bright field images of the petiole of a non-treated leaf adjacent to the leaf treated with SLNs, after 24 h of foliar application. (**a**) presents the cross-section and (**b**) presents the longitudinal section. Fluorescein channel is shown on the left and the bright field channel is shown on the right. These images show the presence of SLNs, suggesting the movement of SLNs through the vascular tissues.

Since the movement of SLNs through the vascular system is required for effective treatment, fluorescence images of the petiole of an adjacent non-treated leaf were analyzed (Fig. 8). Several bright spots could be observed in the Fluorescein channel of the fluorescence images in both cross sections and longitudinal sections. This suggests that some SLNs have moved through the vascular system to the neighboring leaves. Further analysis is required to determine if the SLNs can reach leaves far from the point of application and how long the SLNs are retained in the vascular system.

### MALDI-TOF mass spectrometry

**Fig. 9:**
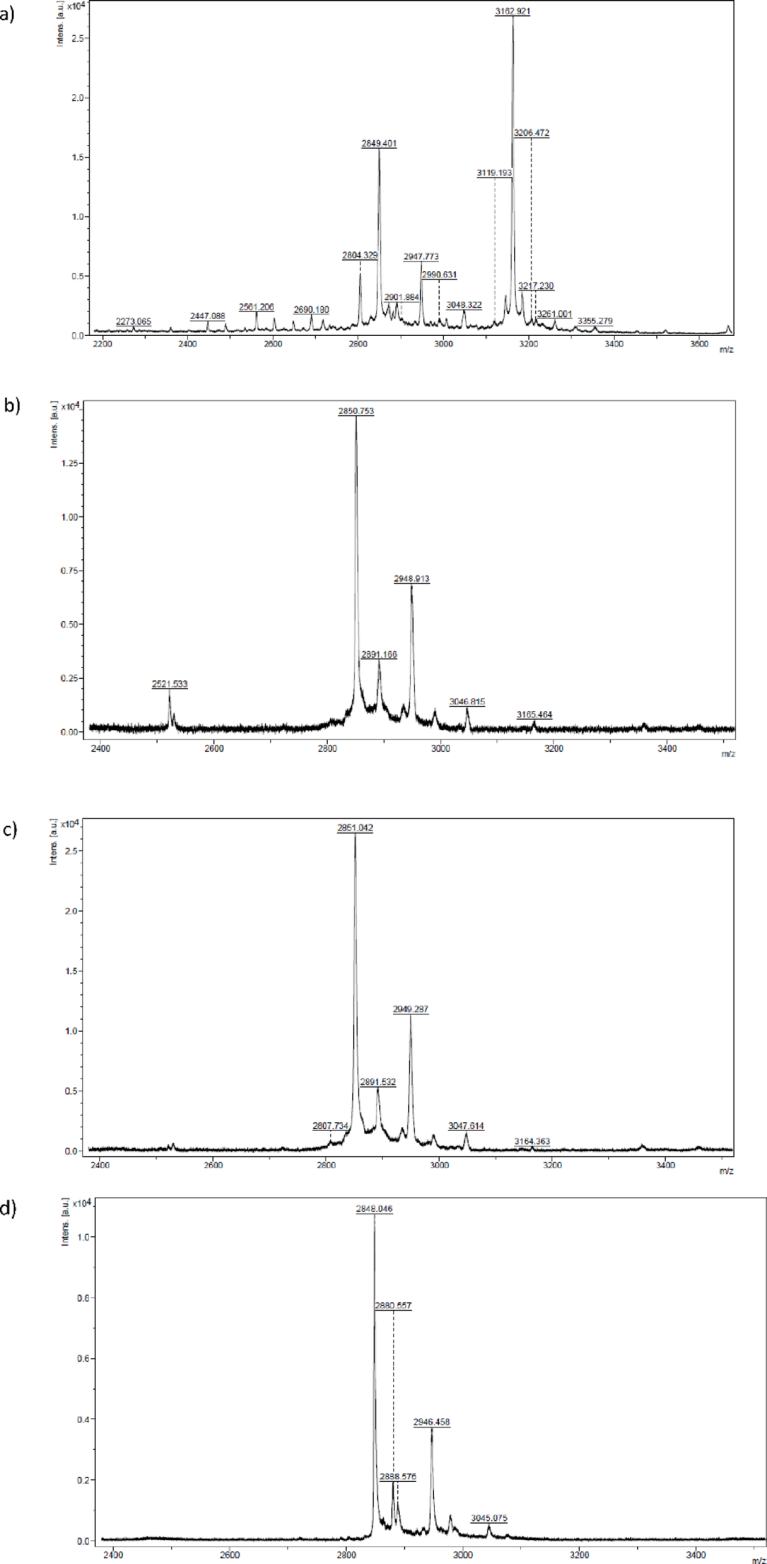
MALDI-MS spectra of (**a**) 400 µg/mL solution of Peptide 57,020, (**b**) SLN-treated leaf extract, (**c**) aqueous peptide-treated leaf extract, and (**d**) untreated leaf extract.

MALDI-TOF mass spectrometry was conducted to verify the presence of peptides within treated leaves. Mass spectra of the treated leaves are presented in Fig. 9. As observed in Fig. 9a, the mass spectrum of the peptide stock solution had a distinct peak at 3163 m/z ratio, which corresponds to the molar mass of Fluorescein-tagged Peptide 57,020. In addition, the peak corresponding to internal standard Melittin could be observed at 2849 m/z ratio^81^. Since the m/z ratios corresponding to the peaks are equal to the molar masses, it indicates that both Peptide 57,020 and Melittin have acquired a single charge during the ionization.

As depicted in Fig. 9b, the spectrum of SLN-treated leaf extract has a small but distinct peak at 3165 m/z ratio, corresponding to Peptide 57,020. It can be observed that this peak is not present in the untreated sample (Fig. 9d), which confirms that this peak actually indicates the presence of Peptide 57,020 in SLN-treated leaf extract. The intensity of this peak is much lower than that of the stock solution, which could result from dilution of the peptide solution in the cellular environment, limitations in peptide uptake, and loss of peptide during the extraction process. As observed in Fig. 9c, the aqueous peptide-treated sample also has a slight peak corresponding to Peptide 57,020. Visual observation of mass spectra reveals that the SLN-treated leaf extract has a larger peak corresponding to Peptide 57,020, compared to the aqueous peptide-treated leaf, suggesting that encapsulation within SLNs can enhance peptide delivery into plant tissues.

Concentration of peptide 57,020 was quantified by analyzing the peaks corresponding to Peptide 57,020 and internal standard in MS spectra, which is presented in Table 3. Spectra of SLN-treated samples demonstrated higher relative peak intensity and relative peak area for Peptide 57,020, indicating that SLN-treated leaves have a higher concentration of Peptide 57,020. Welch’s t-test confirmed that the difference between peak areas for SLN-treated samples and aqueous peptide-treated samples was statistically significant at a 90% confidence level (*p* = 0.0886), which confirms enhanced peptide delivery.

**Table 3:** MS peak data corresponding to Peptide 57,020 in SLN-treated and aqueous peptide-treated leaf extracts.

Relative peak intensity of Peptide 57,020	SLN treated	Aqueous peptide-treated
	(2.76 ± 1.32) * 10^− 2^	(7.91 ± 5.06) * 10^− 3^
Relative peak area of peptide 57,020	(1.41 ± 0.07) * 10^− 2^	(2.44 ± 2.37) * 10^− 3^

#### Evaluation of phytotoxicity

**Fig. 10:**
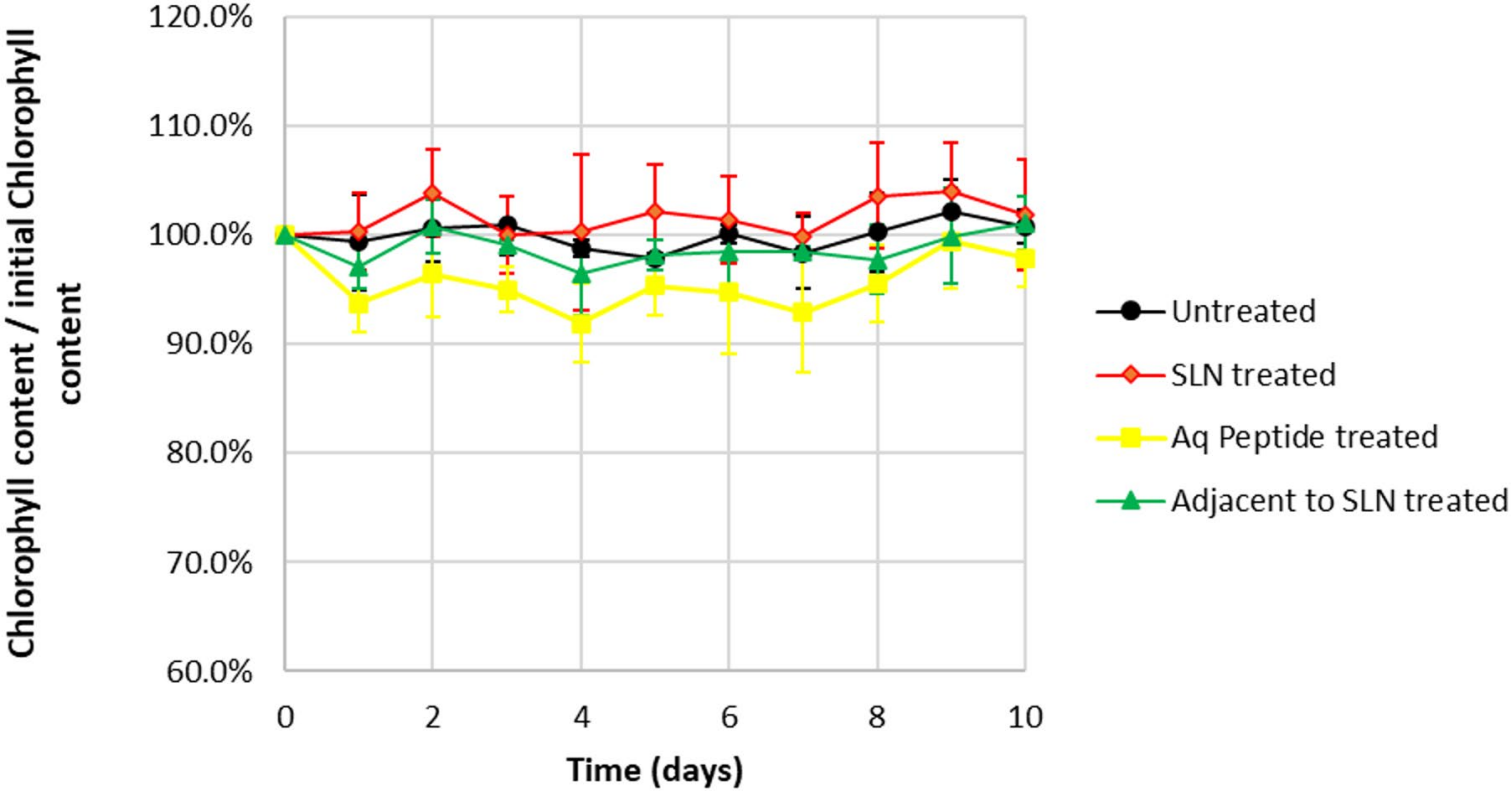
Variation of Chlorophyll content with time for leaves treated with different formulations. Measurements were taken from three leaves per treatment at two locations in each leaf.

Chlorophyll content of treated leaves was monitored following the application of SLNs and aqueous peptides, since reduced Chlorophyll content is usually associated with phytotoxic effects^82^. The variation of Chlorophyll content with time for leaves treated with different formulations is given in Fig. 10. As observed in Fig. 10, SLN-treated leaves did not show any noticeable reduction in Chlorophyll content. Aqueous peptide-treated leaves showed a slight reduction in Chlorophyll content, but it was also less than a 10% reduction compared to the untreated leaves. Statistical analysis revealed that the change in Chlorophyll content between SLN-treated and untreated leaves is not statistically significant (*p* = 0.1268), suggesting that SLNs cause no significant phytotoxicity.

## Conclusions

One of the main challenges in treating citrus greening diseases is the delivery of therapeutic agents to the vascular tissues where the CLas bacteria reside. This study was conducted to evaluate the feasibility of using poly(DADMAC) incorporated nanoparticles to deliver peptides into the vascular tissues of citrus plants. A clear relationship could be observed between poly(DADMAC) concentration and the zeta potential of SLNs, with all formulations including poly(DADMAC) showing positive zeta potential. This is desirable for drug delivery, since SLNs with positive charges can interact with the negatively charged cell surface, and enhance the permeability of plant cells to the therapeutic agents. Fluorescence images of vascular tissues of leaves treated with peptide-encapsulated SLNs and aqueous peptides revealed greater fluorescence in samples treated with SLNs, suggesting that encapsulation within SLNs can enhance the delivery of peptides into the vascular system. Overall, this can be considered as an effective and convenient technique to deliver therapeutics against citrus greening disease, and further developments can be made to enhance the phase stability of SLNs to make it more suitable for practical field applications. Additionally, evaluating the biological or therapeutic effect of the delivered peptide on infected citrus tissues is necessary to determine the functional efficacy of SLNs, and will be a focus of future studies.

## Supplementary Information

Below is the link to the electronic supplementary material.

Supplementary Material 1

## Data Availability

All relevant data is provided within the manuscript or supplementary information files. Any additional information is available upon request.
