# Supplementary material for: Poly(DADMAC) incorporated lipid nanoparticles enhance the delivery of antimicrobial peptides into plant cells
Source: Sci Rep. 2026 May 13;16:21970. doi: 10.1038/s41598-026-52008-6 (PMC13365570; doi:10.1038/s41598-026-52008-6)
Supplement: Supplementary file 1 — Supplementary Material 1 [file 41598_2026_52008_MOESM1_ESM.docx]

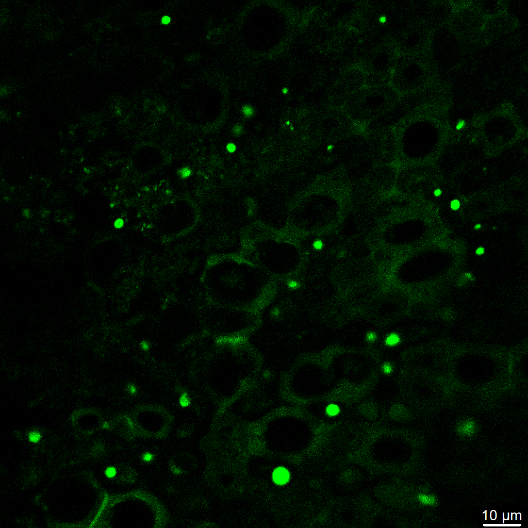

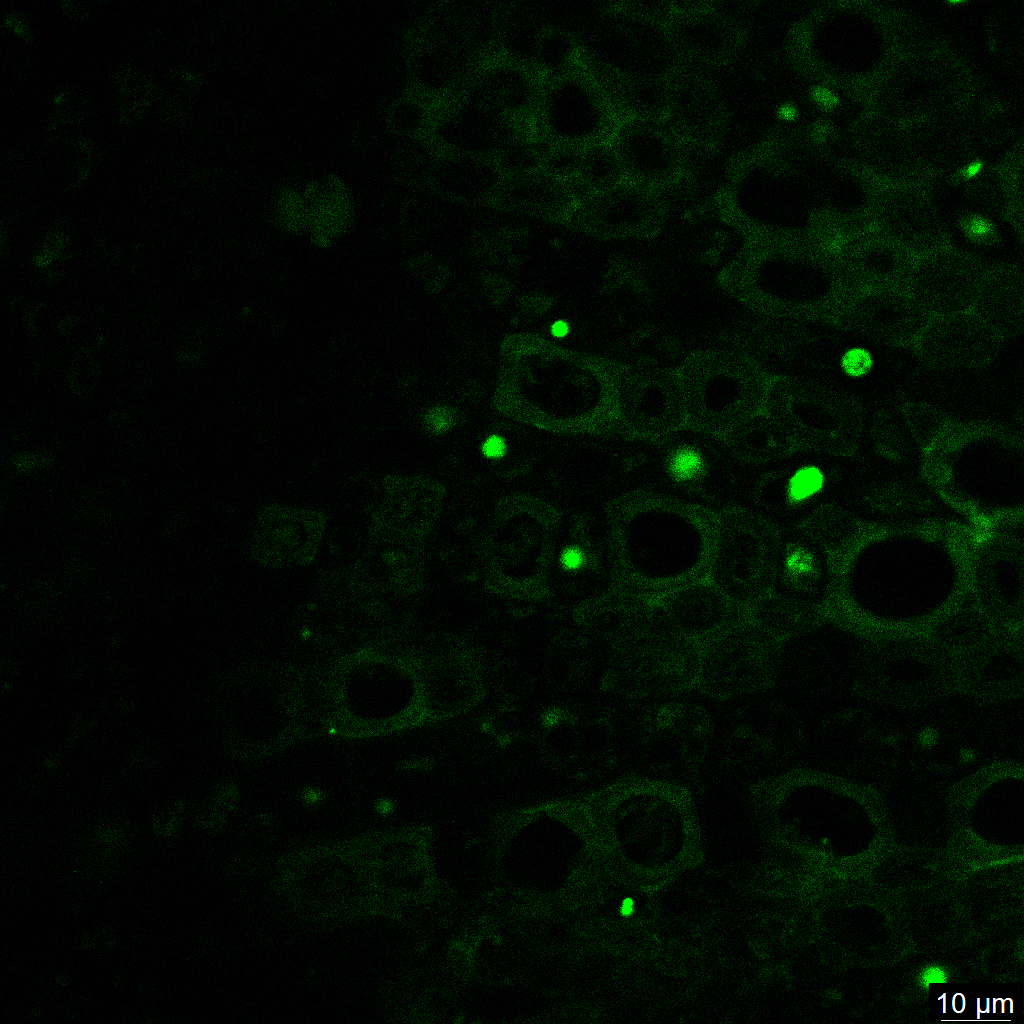


SLN treated – Sample 1

SLN treated – Sample 2

SLN treated – Sample 3

SLN treated – Sample 4


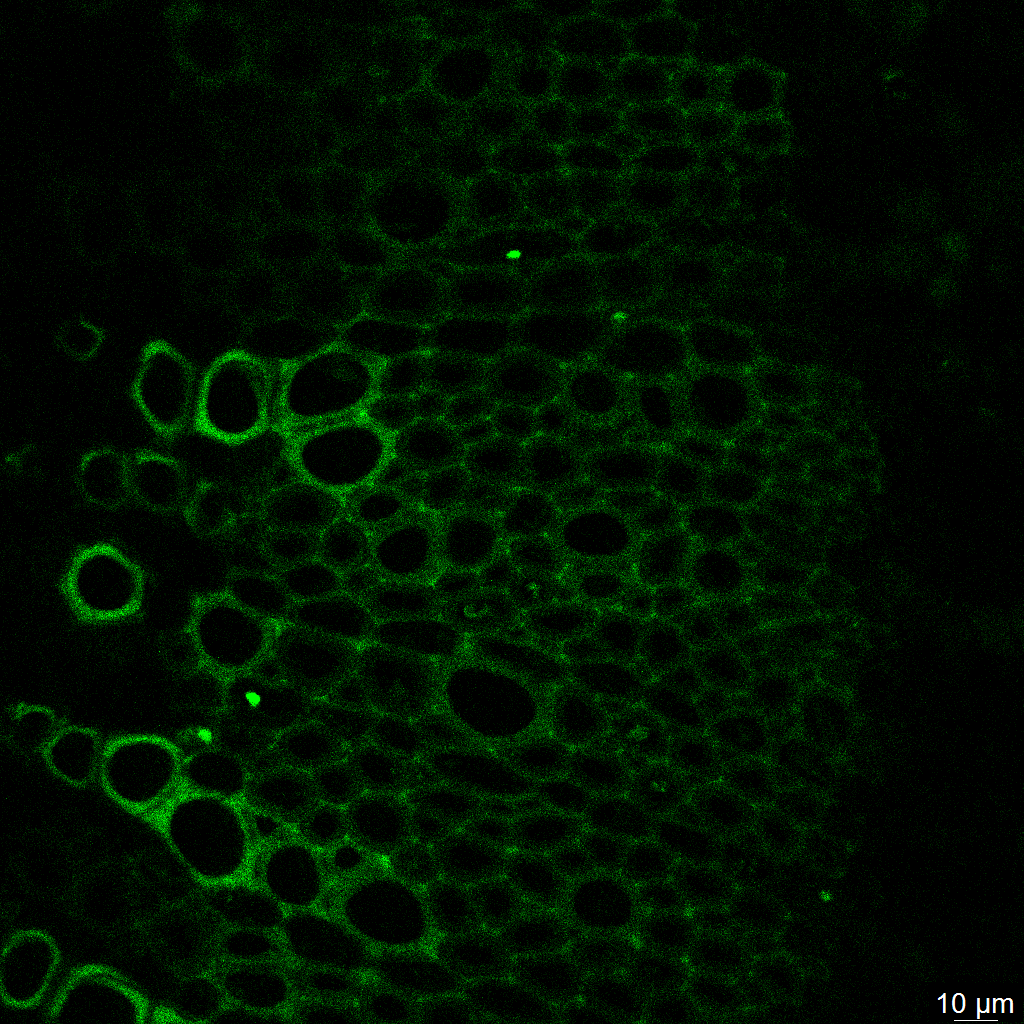

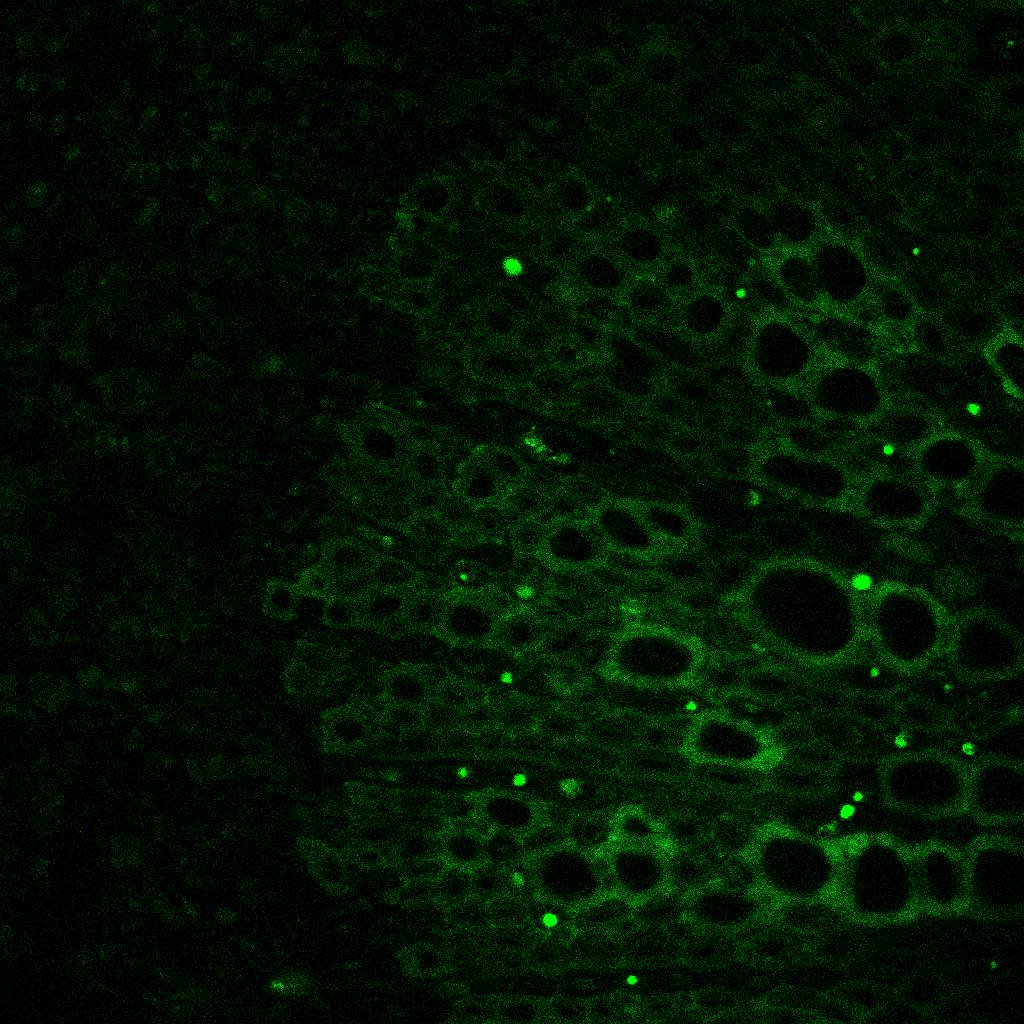

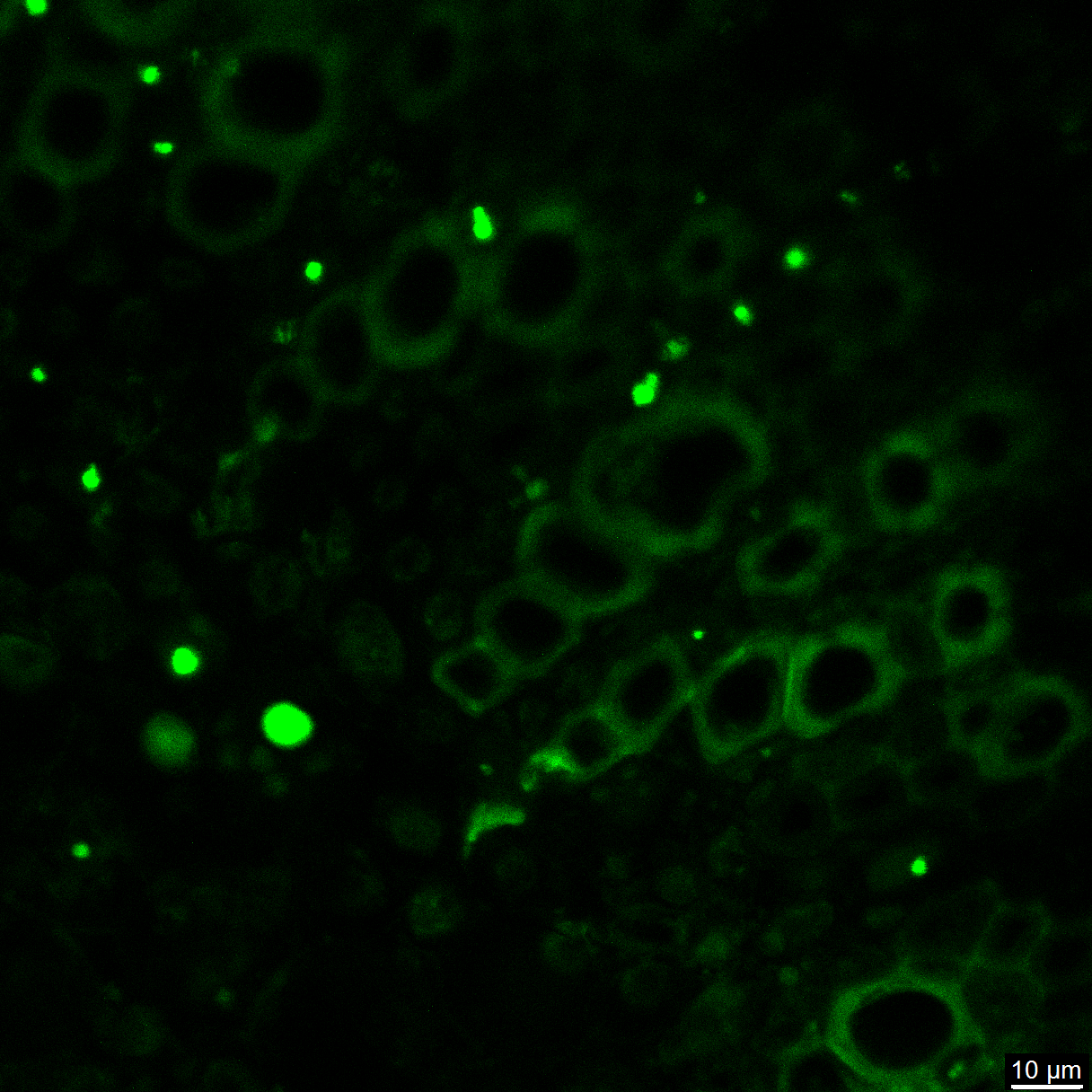


SLN treated – Sample 5

SLN treated – Sample 6


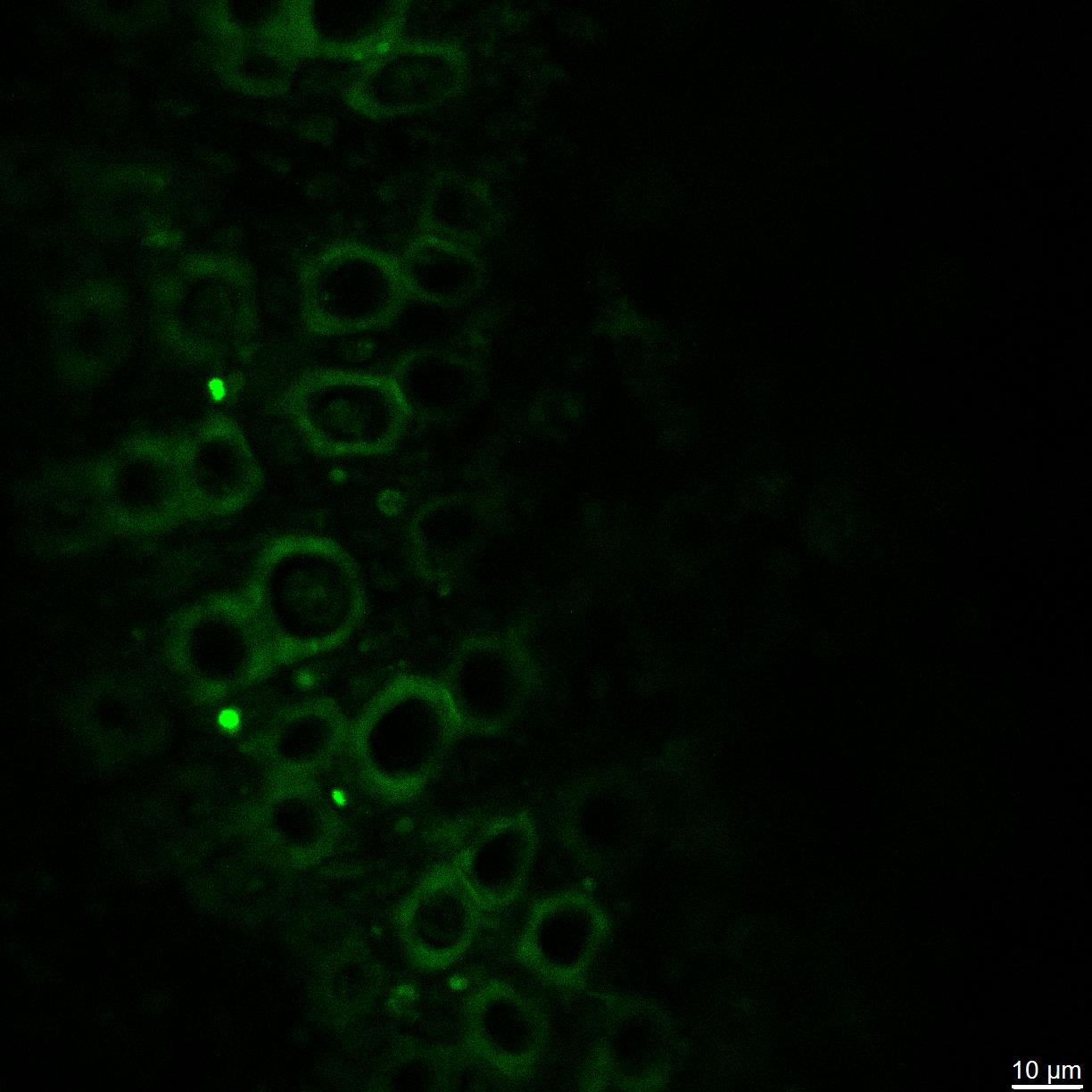

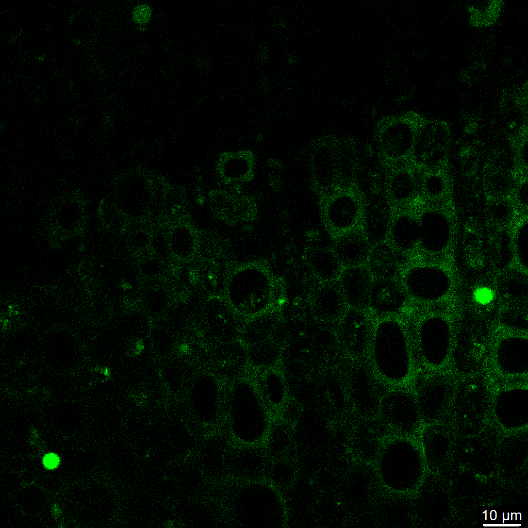

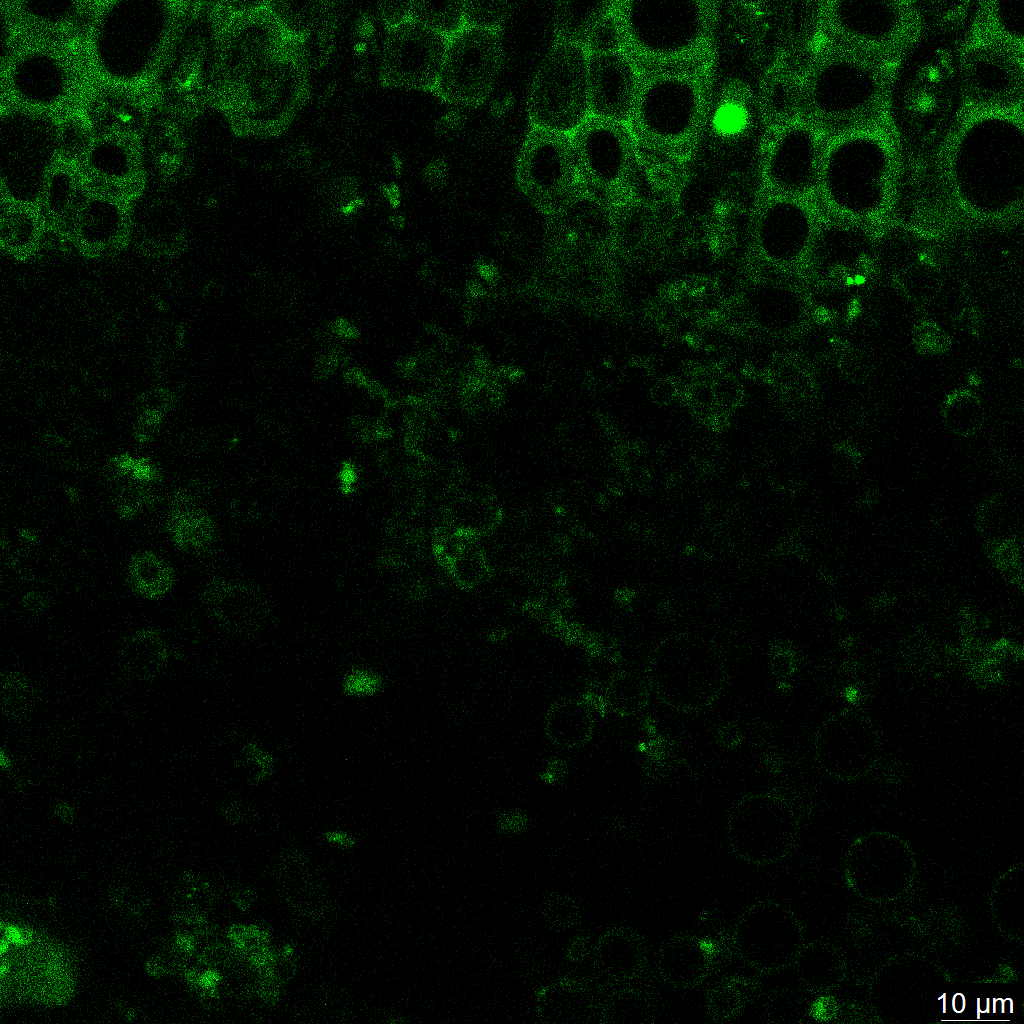


Aqueous peptide treated – Sample 1

Aqueous peptide treated – Sample 2

Aqueous peptide treated – Sample 3

Aqueous peptide treated – Sample 4


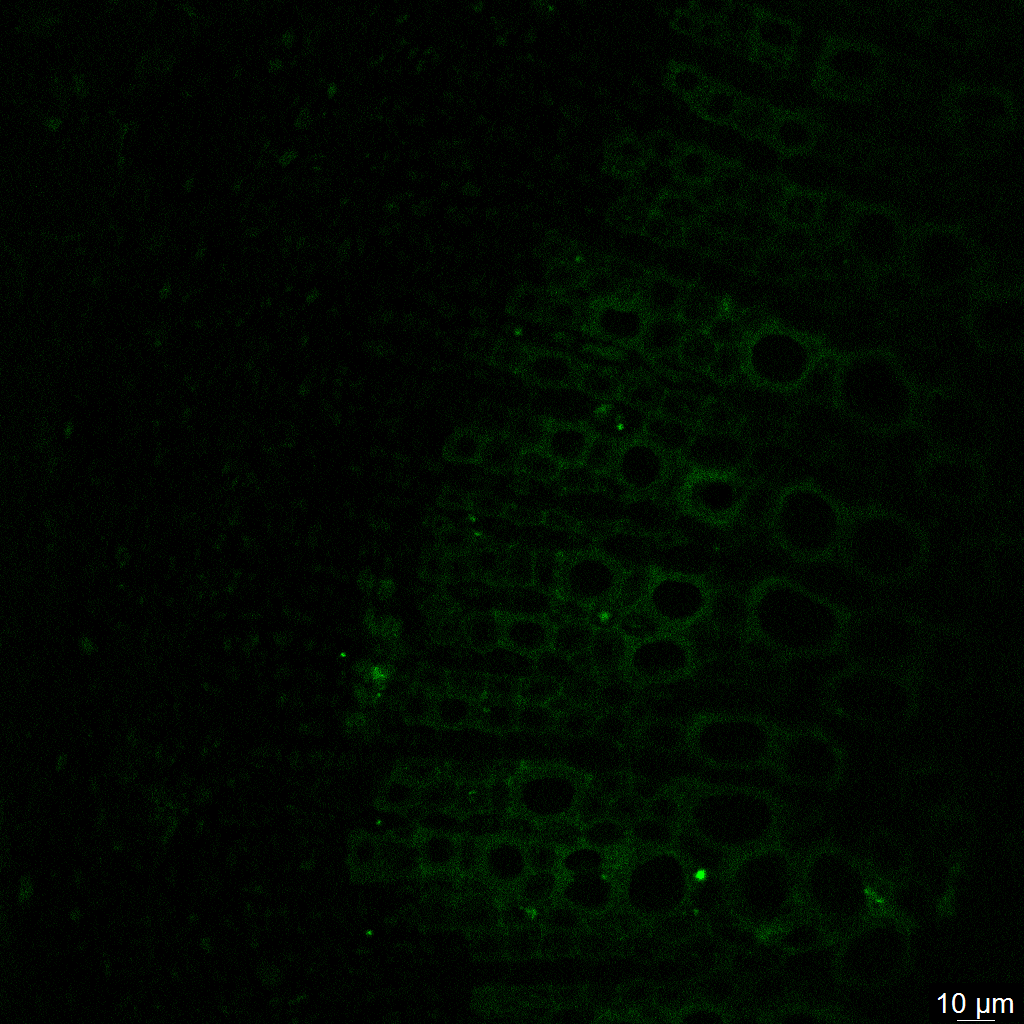

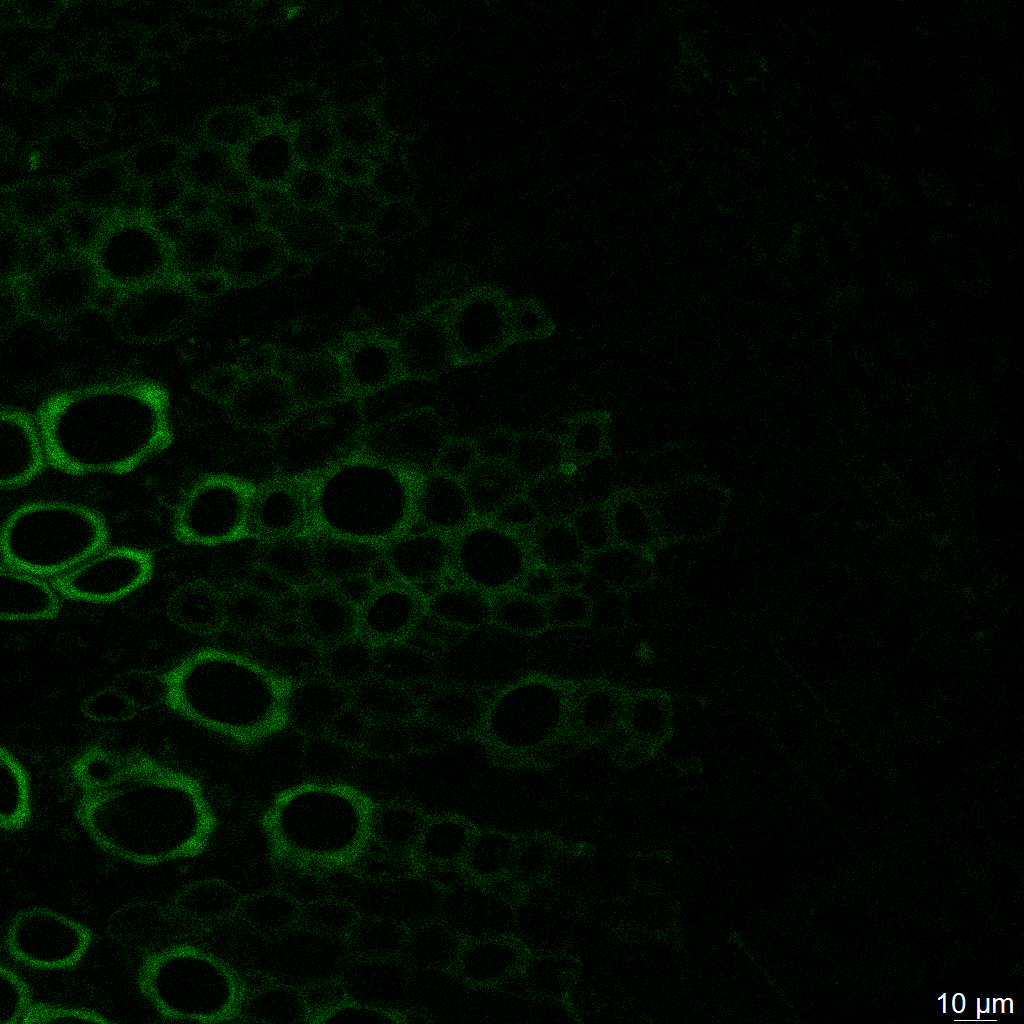

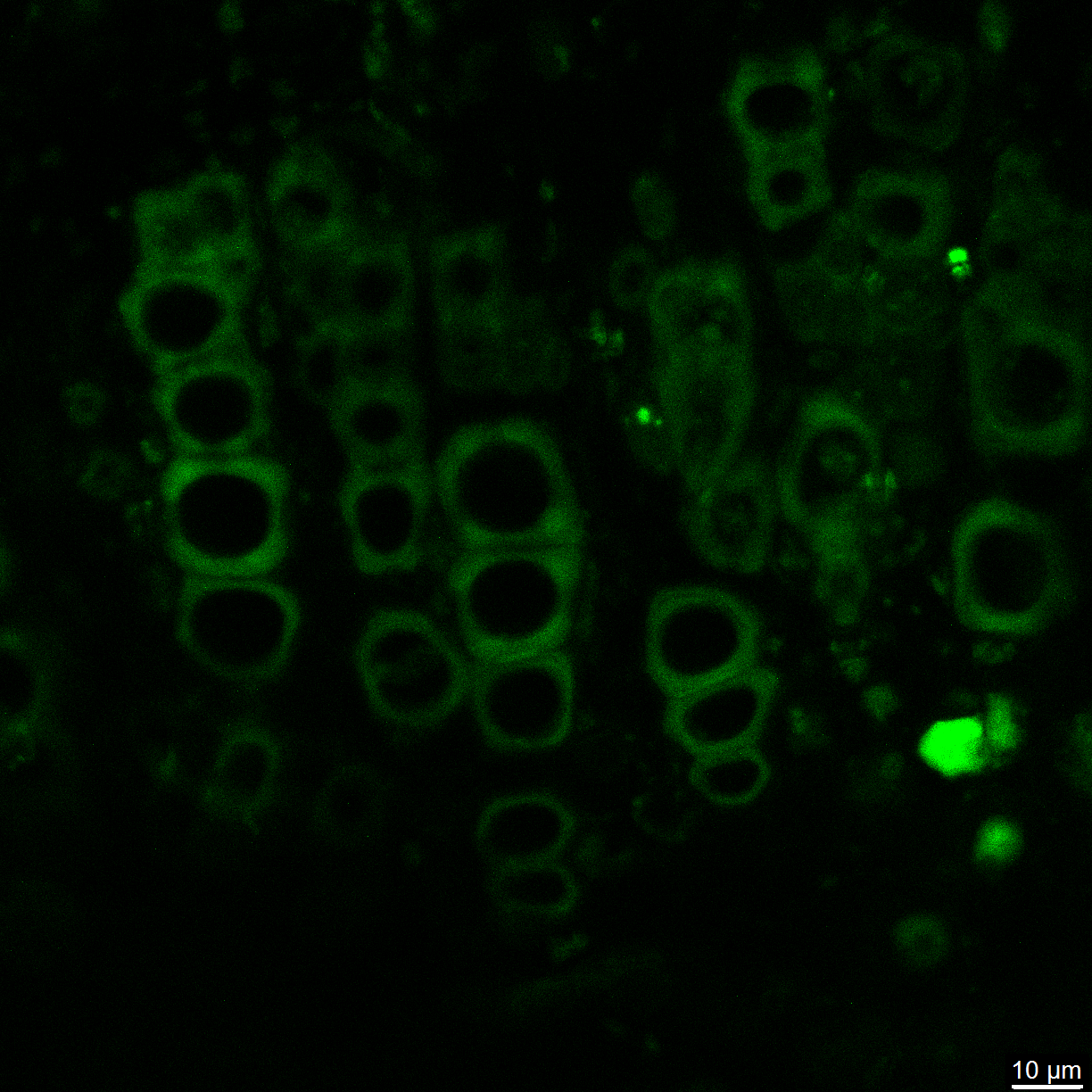


Aqueous peptide treated – Sample 5

Aqueous peptide treated – Sample 6


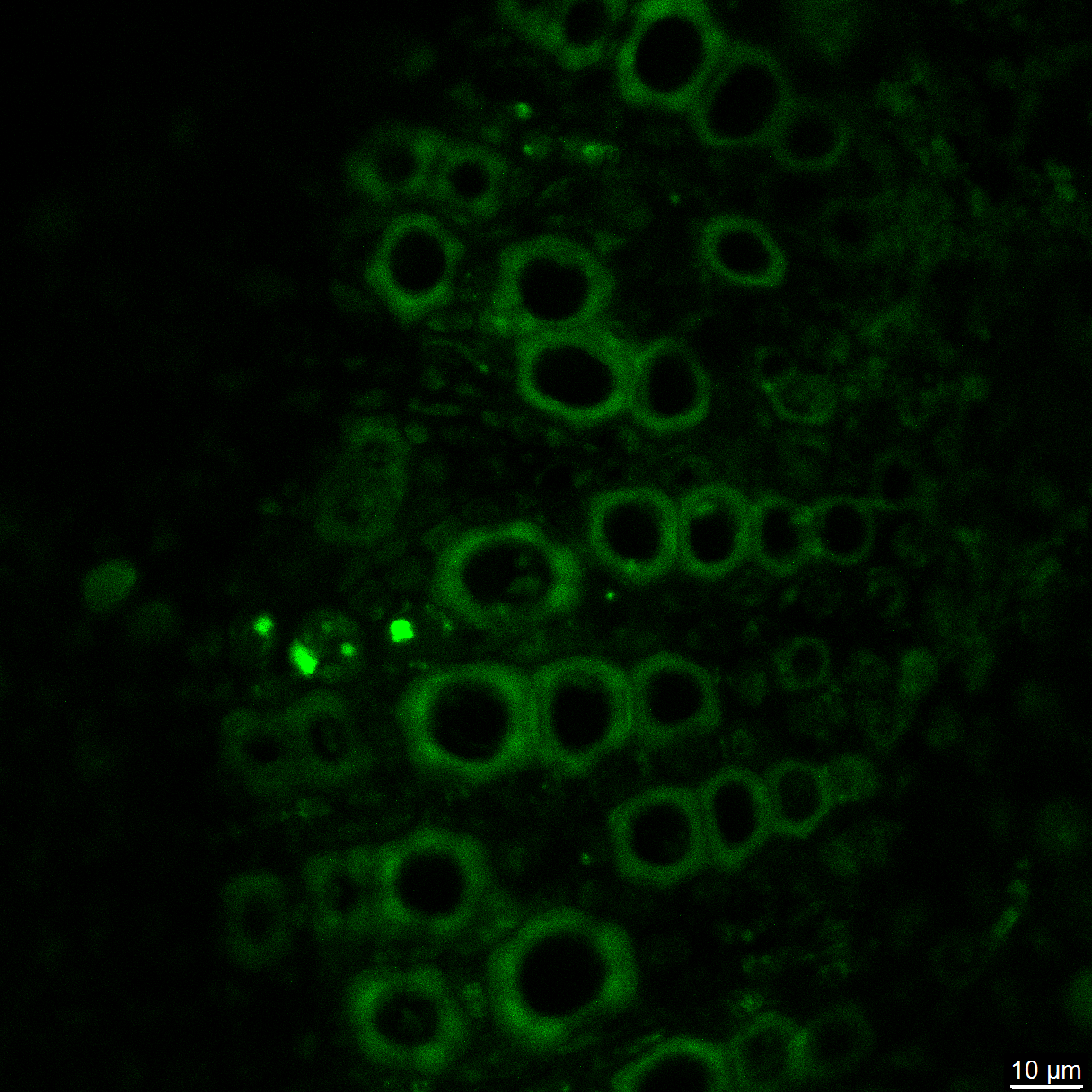

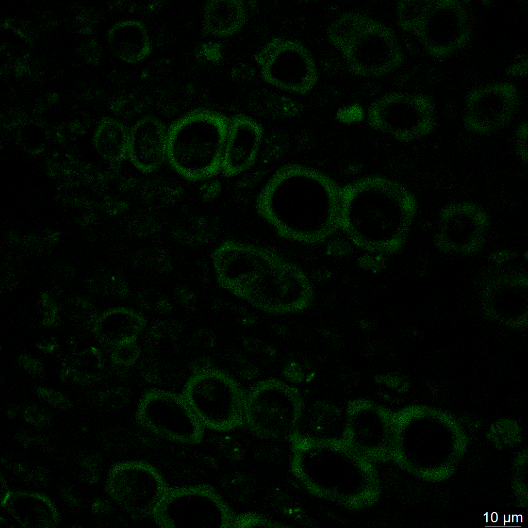

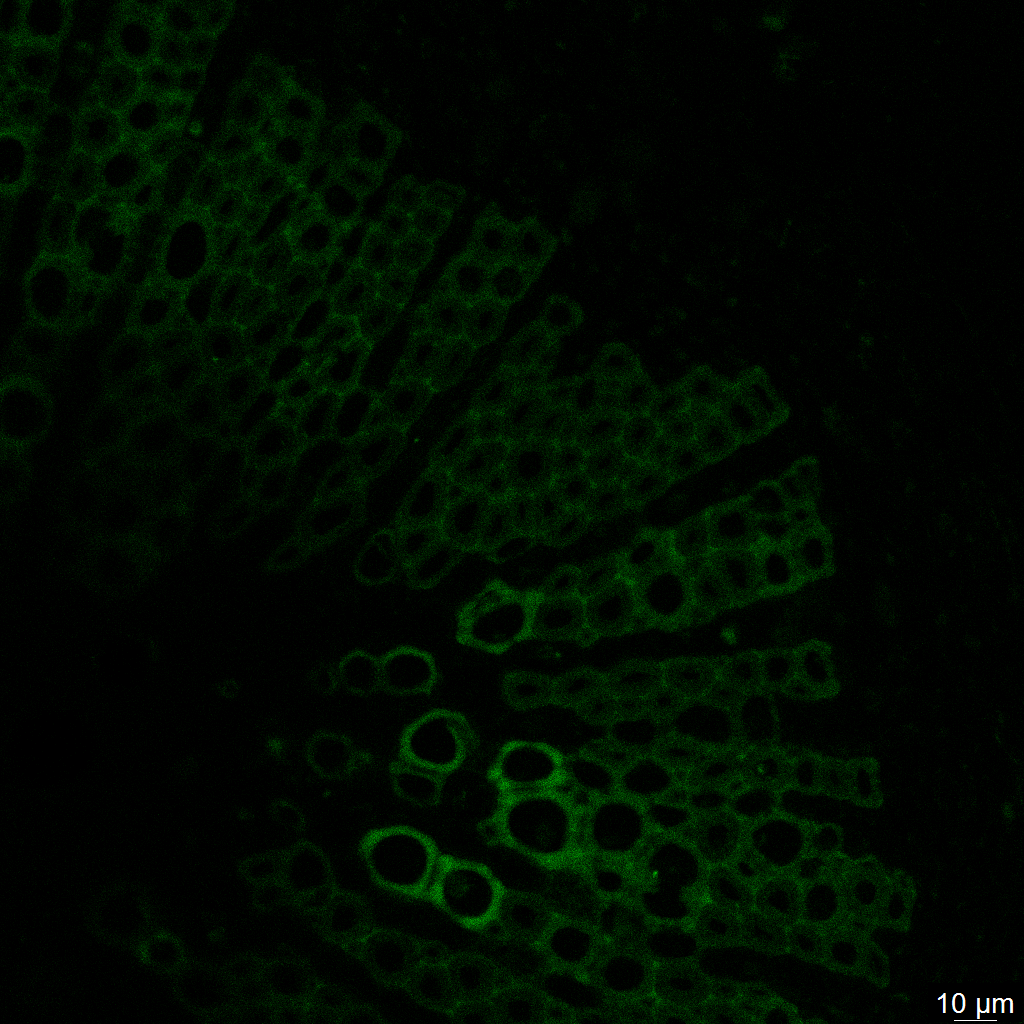


Untreated – Sample 1

Untreated – Sample 2

Untreated – Sample 3


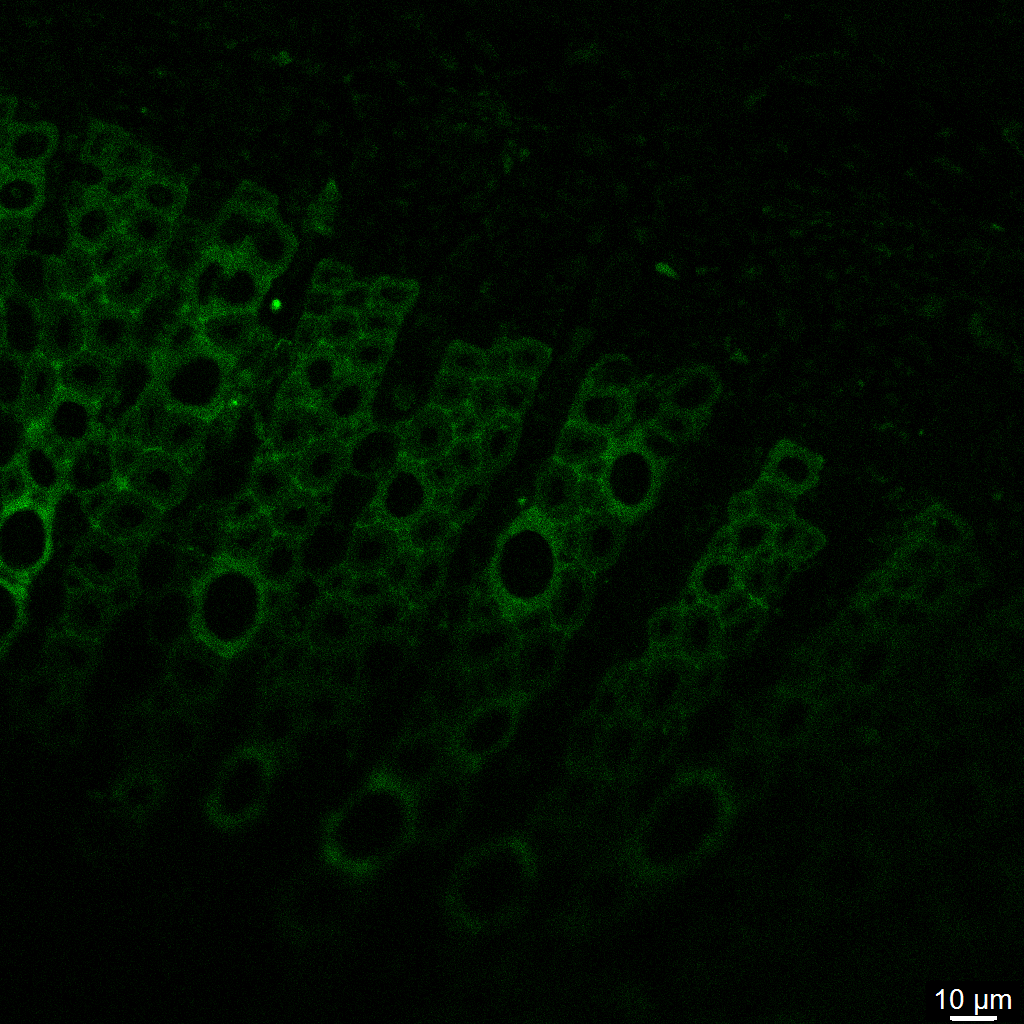


**Figure S1 – Images used to calculate fluorescent parameters**

**Table S1 – Fluorescent parameters of each individual image used to calculate average parameters**

| **Sample** | **Effective area** | **Mean Fluorescence** | **Particle count** | **Particle density** |
| --- | --- | --- | --- | --- |
| Untreated – Sample 1 | 202.272 | 9.633 | 17797 | 87.98548489 |
| Untreated – Sample 2 | 202.272 | 13.058 | 11299 | 55.86042557 |
| Untreated – Sample 3 | 202.272 | 12.517 | 16297 | 80.56972789 |
| SLN treated – Sample 1 | 202.272 | 15.194 | 29313 | 144.9187233 |
| SLN treated – Sample 2 | 160.79 | 14.22 | 20605 | 128.1485167 |
| SLN treated – Sample 3 | 202.272 | 10.908 | 21789 | 107.7212862 |
| SLN treated – Sample 4 | 156.642 | 16.159 | 24577 | 156.8991714 |
| SLN treated – Sample 5 | 285.235 | 14.05 | 18092 | 63.42840114 |
| SLN treated – Sample 6 | 190.781 | 19.722 | 18390 | 96.39324671 |
| Aq peptide treated – Sample 1 | 202.272 | 10.756 | 25899 | 128.0404604 |
| Aq peptide treated – Sample 2 | 138.074 | 12.639 | 14261 | 103.2851949 |
| Aq peptide treated – Sample 3 | 144.79 | 12.775 | 25930 | 179.0869535 |
| Aq peptide treated – Sample 4 | 144 | 12.717 | 16201 | 112.5069444 |
| Aq peptide treated – Sample 5 | 289 | 14.594 | 15409 | 53.3183391 |
| Aq peptide treated – Sample 6 | 285.235 | 16.505 | 18275 | 64.06997739 |

**Table S2 – Effect test results on the impact of Tween(80) and poly(DADMAC) concentrations on particle size and zeta potential.**

| **Factor** | **Number of levels** | **Degrees of freedom** | **Sum of squares** | **F ratio** | **p-value** |
| --- | --- | --- | --- | --- | --- |
| For particle size | | | | | |
| Tween 80% | 3 | 2 | 3527079.0 | 9.6304 | 0.0012 |
| Poly(DADMAC) % | 3 | 2 | 9108074.7 | 24.8688 | <0.0001 |
| Tween 80% × poly(DADMAC) % | 9 | 3 | 1617616.7 | 2.9445 | 0.0578 |
| For zeta potential | | | | | |
| Tween 80% | 3 | 2 | 678.4293 | 2.5214 | 0.1056 |
| Poly(DADMAC) % | 3 | 2 | 1019.7689 | 3.7900 | 0.0402 |
| Tween 80% × poly(DADMAC) % | 9 | 3 | 659.2683 | 1.6335 | 0.2133 |

**Table S3 – Welch’s t-test results for the fluorescent intensity analysis presented in Table 2**

|  | **For corrected mean fluorescence** |
| --- | --- |
| Mean (SLN-treated) | 1.596 |
| Mean (Aq peptide-treated) | 1.155 |
| Standard deviation (SLN-treated) | 0.723 |
| Standard deviation (Aq peptide-treated) | 1.326 |
| Sample size (SLN-treated) | 6 |
| Sample size (Aq peptide-treated) | 6 |
| T-statistic | -0.715 |
| Degrees of freedom | 7.73 |
| P-value | 0.4959 |

**Table S4 – Welch’s t-test results for the MALDI-MS analysis presented in Table 3**

|  | **For peak intensity** | **For peak area** |
| --- | --- | --- |
| Mean (SLN-treated) | 0.027614 | 0.014132 |
| Mean (Aq peptide-treated) | 0.007908 | 0.002444 |
| Standard deviation (SLN-treated) | 0.013219 | 0.006979 |
| Standard deviation (Aq peptide-treated) | 0.005064 | 0.002372 |
| Sample size (SLN-treated) | 3 | 3 |
| Sample size (Aq peptide-treated) | 3 | 3 |
| T-statistic | 2.4112 | 2.7466 |
| Degrees of freedom | 2.5747 | 2.4557 |
| P-value | 0.1091 | 0.0886 |
